# Dynamic Associations of Milk Components With the Infant Gut Microbiome and Fecal Metabolites in a Mother–Infant Model by Microbiome, NMR Metabolomic, and Time-Series Clustering Analyses

**DOI:** 10.3389/fnut.2021.813690

**Published:** 2022-01-07

**Authors:** Yosuke Komatsu, Daiki Kumakura, Namiko Seto, Hirohisa Izumi, Yasuhiro Takeda, Yuki Ohnishi, Shinji Nakaoka, Tomoyasu Aizawa

**Affiliations:** ^1^Graduate School of Life Science, Hokkaido University, Sapporo, Japan; ^2^Health Care and Nutritional Science Institute, Morinaga Milk Industry Co. Ltd., Zama, Japan; ^3^Center for Food and Medical Innovation Promotion, Institute for the Promotion of Business-Regional Collaboration of Hokkaido University, Sapporo, Japan; ^4^Department of Advanced Transdisciplinary Science, Faculty of Advanced Life Science, Hokkaido University, Sapporo, Japan

**Keywords:** NMR metabolomics, time-series clustering analysis, breast milk, bioactive component, human milk oligosaccharide, infant gut microbiome, infant fecal metabolites

## Abstract

**Background:** The gut microbiome and fecal metabolites of breastfed infants changes during lactation, and are influenced by breast milk components. This study aimed to investigate dynamic associations of milk components with the infant gut microbiome and fecal metabolites throughout the lactation period in a mother–infant model.

**Methods:** One month after delivery, breast milk and subsequent infant feces were collected in a pair for 5 months from a mother and an exclusively breastfed infant. Composition of the fecal microbiome was determined with 16S rRNA sequencing. Low-molecular-weight metabolites, including human milk oligosaccharides (HMOs), and antibacterial proteins were measured in feces and milk using ^1^H NMR metabolomics and enzyme-linked immunosorbent assays. The association of milk bioactive components with the infant gut microbiome and fecal metabolites was determined with Python clustering and correlation analyses.

**Results:** The HMOs in milk did not fluctuate throughout the lactation period. However, they began to disappear in infant feces at the beginning of month 4. Notably, at this time-point, a bifidobacterium species switching (from *B. breve* to *B. longum* subsp. *infantis*) occurred, accompanied by fluctuations in several metabolites including acetate and butyrate in infant feces.

**Conclusions:** Milk bioactive components, such as HMOs, might play different roles in the exclusively breastfed infants depending on the lactation period.

## Introduction

The gut microbiome plays an important role in the normal growth and development of infants, and it is less diverse and biased toward pathogens in premature infants with necrotizing enterocolitis (1). Perturbation of the gut microbiome could enhance the inflammatory response and lead to the development of necrotizing enterocolitis in infants (2). In contrast, the gut microbiome of healthy infants delivered at full term has a relatively high proportion of the genus *Bifidobacterium*, which confers benefits, such as the prevention of bacterial infections and the maturation of immune functions, to infants (3). In addition to *Bifidobacterium*, anaerobic bacteria such as *Bacteroides* and *Clostridium* have been identified as colonizers during the first 6 months, and are known to have various effects on the development and maturation of infants (4). Generally, bacterial families in exclusively breastfed infants have a higher occupancy of *Bifidobacteriaceae* and a lower occupancy of *Enterococcaceae* and *Enterobacteriaceae* than those in formula-fed infants (5). The infant gut microbiome changes during lactation, and the contents of bacterial genera such as *Bifidobacterium* and *Lactobacillus* fluctuate dramatically even in exclusively breastfed infants (6, 7). Furthermore, the dominant bacterial species are entirely different at different stages of lactation in breastfed infants (8, 9). This suggests that occupancy of the infant gut microbiome continues to change as the abundance of each bacterial species increases or decreases individually during the lactation period. These changes in the gut microbiome could be influenced by the maturation of gastrointestinal function in infants and by the composition of the breast milk ingested by infants. However, because previous studies were based on monthly sampling (6–9), the detailed time-course changes in the gut microbiome, which could be affected by the components in the ingested breast milk, remain unclear.

Breastfeeding is recommended by the World Health Organization and numerous pediatric associations as the best way of feeding infants during at least 6 months of life; breastfeeding is extremely beneficial for the health and development of infants (10). In fact, breast milk, a dynamic biofluid, is recognized as the only food able to meet all of a new-born's nutritional needs. Moreover, it includes various bioactive compounds, such as immunoglobulins (Igs), cytokines, and growth factors, important to prevent infection and promote the development of organs and systems (e.g., the immune system) (11, 12). Additionally, low-molecular-weight metabolites, up to a molecular size of ~1500 Da, are also contained in breast milk. Recent studies have reported that breast milk components are affected by several factors, including diet, genetic background, lifestyle, and body mass index of the mother, among which the lactation stage has been reported to be the most influential (13, 14). Some of fluctuating milk components are bioactive and affect the gut microbiome of infants. For example, human milk oligosaccharides (HMOs) are unconjugated glycans with a lactose core varying in chain length from 3 to 15 carbohydrates and are assimilated by specific bacteria (15). Fucosylated lactoses such as 2′-fucosyllactose (2′-FL) and 3-FL, sialylated lactoses such as 3′-sialyllactose (3′-SL) and 6′-SL, and oligosaccharides with lacto-*N*-biose structure such as lacto-*N*-tetraose (LNT) and lacto-*N*-fucopentaose (LNFP) I are abundant HMOs in breast milk. These HMOs might play essential roles in supporting the bifidobacteria-predominant gut microbiome in breastfed infants (16). Furthermore, breast milk contains antibacterial proteins such as IgA, lactoferrin (LF), and lysozyme (LYZ), which can contribute to establishing the gut microbiome of infants fed breast milk (17). The gut microbiome modified by bioactive milk components confers various benefits to infants via its metabolites. In fact, a study using an animal model has reported that bifidobacteria could inhibit *Escherichia coli* infection via the production of acetate and the improvement of the intestinal barrier, due to the use of indigestible oligosaccharides (18). In addition, short-chain fatty acids (SCFAs) produced by the gut microbiome have been reported to increase resistance to inflammation and promote sympathetic nervous system maturation (19). However, the relationship between bioactive milk components, such as HMOs and antibacterial proteins, and the gut microbiome and fecal metabolites of breastfed infants remains unclear. This study aimed to clarify whether the infant gut microbiome and fecal metabolites are associated with milk components throughout the lactation period in a mother–infant model using microbiome analysis, NMR metabolomics, time-series clustering, and Spearman correlation analyses. In this study, biological samples were collected approximately every 4–6 days from one mother and an exclusively breastfed infant. Although this study is based on one mother–infant model and is preliminary, it is the first to examine changes in the infant gut microbiome and fecal metabolites weekly over the entire lactation period. Although there exists an omics study of breast milk and infant feces in one mother-infant pair, they have confirmed the influence of complementary foods during the weaning period (20). To our knowledge, there are no studies that have followed the detailed time-course changes in breast milk and an exclusively breastfed infant's feces during the lactation period. Components in breast milk collected weekly to correspond with the sampling time of infant feces were also analyzed. By analyzing these cross-data obtained frequently, dynamic associations between breast milk and infant feces could be determined, and a hypothesis to be confirmed in a large-scale clinical trial would be proposed. We believe that establishing interactions between milk components and fecal metabolites in the gut microbiome will facilitate a better understanding of how breastfeeding affects infant development, as well as the design of nutritional and functional foods for infants at various stages of lactation.

## Materials and Methods

### Subject and Sample Collection

The institutional review board at the Japan Conference of Clinical Research approved this study (approval number: BONYU-01), which proceeded in accordance with the latest Declaration of Helsinki in 2013. The healthy mother of a new-born infant provided written, informed consent for the participation of herself and her infant in all procedures associated with the study. One month after birth, mature breast milk samples were manually by hand-stimulation of the breast collected from one breastfeeding mother once every 4–6 days for 5 months. Breast milk sampling intervals varied between months: 9 times during Month 1 (Day 34–59), 8 times during Month 2 (Day 61–82), 7 times during Month 3 (Day 99–118), 8 times during Month 4 (Day 122–148), and 6 times during Month 5 (Day 155–163). Then, whenever the infant of the mother was breastfed, the first excreted feces sample was collected. This covered the whole lactation period until just before complementary food was introduced. Of note, the infant was exclusively breastfed during the experimental period. The collected samples were temporarily stored at −20°C until 1 month and were subsequently transferred to −80°C storage conditions until analysis.

### DNA Extraction

Total DNA was extracted from the feces samples, as described previously (21). Briefly, 200 μL of fecal sample in GuSCN solution was lysed with glass beads (300 mg, 0.1 mm diameter) and 300 μL of lysis buffer (No. 10 buffer, Kurabo Industries Ltd., Osaka, Japan) with a FastPrep-245G (MP Biomedicals LLC, Santa Ana, CA, USA) at a 5 power level for 45 s with 5 min cooling intervals on ice. After centrifugation at 12,000 rpm for 5 min, DNA was extracted from 200 μL of the supernatant using the GENE PREP STAR PI-480 (Kurabo Industries Ltd.) following the manufacturer's protocol.

### Microbiome Analysis

After DNA extraction, PCR amplification and DNA sequencing of the V3-V4 region of the bacterial 16S rRNA gene was performed in an Illumina MiSeq instrument (Illumina, San Diego, CA, USA) as described previously (21).

### Real-Time PCR

Real-time PCR was performed using an ABI PRISM 7500 fast real- time PCR system (Thermo Fisher Scientific, MA, USA) with TB Green^®^ Premix Ex Taq™ Tli RNaseH Plus (TaKaRa Bio Inc., Kusatsu, Japan) following the manufacturer's protocol. The primers used to amplify the 16S rRNA sequences from *B. breve* were F: 5′-CCGGATGCTCCATCACAC-3′ and R: 5′-ACAAAGTGCCTTGCTCCCT-3′; those used to amplify the 16S rRNA sequences from *B. longum* subsp. *infantis* were F: 5′-GACGAGGAGGAATACAGCAG-3′ and R: 5′-CACGAACAGCGAATCATGGATT-3′; those used to amplify the 16S rRNA sequences from *B. longum* subsp. *longum* were F: 5′-TTCCAGTTGATCGCATGGTC-3′ and R: 5′-GGGAAGCCGTATCTCTACGA-3′. Primers and amplification methods were determined based on previous reports (22, 23). For the determination of bacterial counts, bacterial solutions with already known their counts were used as a standard. All real-time PCR samples were assessed in duplicate.

### Measurement of Macronutrients in Breast Milk

The concentration of fat, protein, carbohydrate, and total solid contents in homogenized human milk samples (3 mL) were measured using the Human Milk Analyzer (MIRIS AB, Uppsala, Sweden) via a medium infrared transmission spectroscopy technique. Before analysis, all milk samples were thawed and homogenized using the ultrasonic MIRIS sonicator (MIRIS AB) and were maintained at 40°C prior to measurements.

### Measurement of Antibacterial Proteins in Breast Milk and Infant Feces

The total IgA, LF, and LYZ levels in breast milk and infant feces were measured using a human enzyme-linked immunosorbent assays (ELISA) kit (Abcam, Cambridge, UK) according to manufacturer's protocols. Briefly, extracts of infant feces were prepared for ELISA using the same method as the preparation for NMR analysis. All samples were diluted to meet the assay range of the ELISA kits. For IgA ELISA, breast milk samples were diluted to 1 × 10^5^, and infant fecal samples were diluted to 2 × 10^4^. For LF ELISA, breast milk samples were diluted to 2 × 10^6^, and infant fecal samples were diluted to 1 × 10^5^. For LYZ ELISA, breast milk samples were diluted to 5 × 10^5^, and infant fecal samples were diluted to 1 × 10^2^. All assays were performed in duplicate.

### Sample Preparation for NMR Analysis

Breast milk samples (1 mL) were centrifuged at 3000 × g for 30 min at 4°C, and the aqueous layer was carefully removed and filtered through a 5 kDa cut-off filter (UltrafreeMC-PLHCC filter; HMT, Tsuruoka, Japan) at 9000 × g at 4°C to remove lipids and proteins. The filtrate (540 μL) was mixed with 60 μL 100% D_2_O (Cambridge Isotope Laboratories, Inc., Andover, MA, USA) containing 500 mM NaP, 5 mM 3-(trimethylsilyl)propionic acid-d4 sodium salt (TSP), and 0.04% (w/v) NaN_3_, and 550 μL of the mixture was transferred to a 5 mm NMR tube (Shigemi, Hachioji, Japan). Fecal extracts were prepared for NMR analysis as follows: fecal samples were mixed with MilliQ water (Millipore, Billerica, MA, USA) containing 50 mM NaP, 0.5 mM TSP, 0.004% (w/v) NaN_3_, and 10% D_2_O (per 100 mg of feces). This mixture was homogenized at 1800 rpm for 15 min at 4°C using a tube mixer (EYELA CM-1000; Wakenyaku, Kyoto, Japan). The shaken samples were then centrifuged at 15,000 × *g* for 10 min at 4°C. The supernatant pH was adjusted to 7.4 ± 0.1 by adding small amounts NaOH or HCl to minimize pH-based peak movement. The 550 μL aliquot was transferred to a 5 mm NMR tube.

### NMR Spectra Acquisition and Spectral Data Processing

^1^H NMR spectra were recorded using a Bruker 600 MHz AVANCE spectrometer (Bruker, Billerica, MA, USA) equipped with a cryoprobe at a proton frequency of 600.13 MHz with the sample temperature controlled at 298 K. The acquisition conditions and data processing procedures were the same as in a previous report (24). Briefly, ^1^H NMR spectra were recorded using a water-suppressed standard one-dimensional NOESY1D pulse sequence. Each spectrum consisted of 32,768 data points with a spectral width of 12 ppm. The acquisition time was 2.28 s, and the number of scans was 128. A water-suppressed pulse sequence was used to reduce the residual water signal at the water frequency with a recycle delay [D1 (Bruker notated)] of 2.72 s and a mixing time [D8 (Bruker notated)] of 0.10 s. A 90° pulse length was automatically calculated in the analysis of each sample. All raw spectra were manually corrected for phase and baseline distortions against TSP resonance at δ = 0.0 ppm using the Delta 5.0.4 software (JEOL, Tokyo, Japan) and then analyzed. Spectral binning, multivariate analysis, or direct metabolite relative quantification was performed. The spectra were normalized to the peak area value of the internal standard TSP using the NMR Suite 7.5 Processor (Chenomx, Edmonton, Canada), and the normalized spectral data were further processed. Briefly, in the first round of processing, the 0.0–10.0 ppm chemical shift region was integrated into regions with a width of 0.04 ppm, while the spectral regions related to residual water area (4.68–5.08 ppm) were removed from the multivariate analysis to eliminate the effects of imperfect water suppression. In the second round of processing, metabolite assignment and quantification were determined using the 600 MHz library from Chenomx NMR Suite and previous reports as reference (25–28).

### Statistical Analyses

The spectral data matrix was obtained using SIMCA-P 14.0 software (Umetrics, Umeå, Sweden). Concentrations of breast milk components and binned NMR spectra in infant feces during each lactation period were assessed using principal component analysis (PCA). PCA score plots were obtained to visualize the clustering pattern of the samples in the context of two principal components (PC1 and PC2), with each point denoting an individual sample. For microbiome data, weighted UniFrac distance metrics analysis was performed using operational taxonomic units (OTUs) for each sample. Following PCA, supervised classification of orthogonal partial least squares discriminant analysis (OPLS-DA) with group information was also performed to compare the differences between five groups. PCA and OPLS-DA were conducted according to the distance matrix using SIMCA-P 14.0 software. The α-diversity of the microbiome in feces at various lactation points was represented by the Shannon index. A time-series clustering analysis using Dynamic Time Warping in Python version 3.7.3 was performed to verify the similarity between bioactive milk components, the infant gut microbiome, and fecal metabolites in infant feces. After logarithmizing the numerical data, standardization was performed via Z-scoring with the mean and standard deviation set to 0 and 1, respectively. Furthermore, bioactive milk components, the infant gut microbiome, and fecal metabolites showing similar changes over the lactation period were categorized into the same group using the scientific computing package SciPy and the visualization package Seaborne (29). The association of bioactive milk components with the infant gut microbiome and fecal metabolites was explored using Spearman rank correlation coefficient analyses and heatmaps generated using SciPy and Seaborne.

## Results

### Profiling of Microbiome and Metabolites in Infant Feces

**Figure 1:**
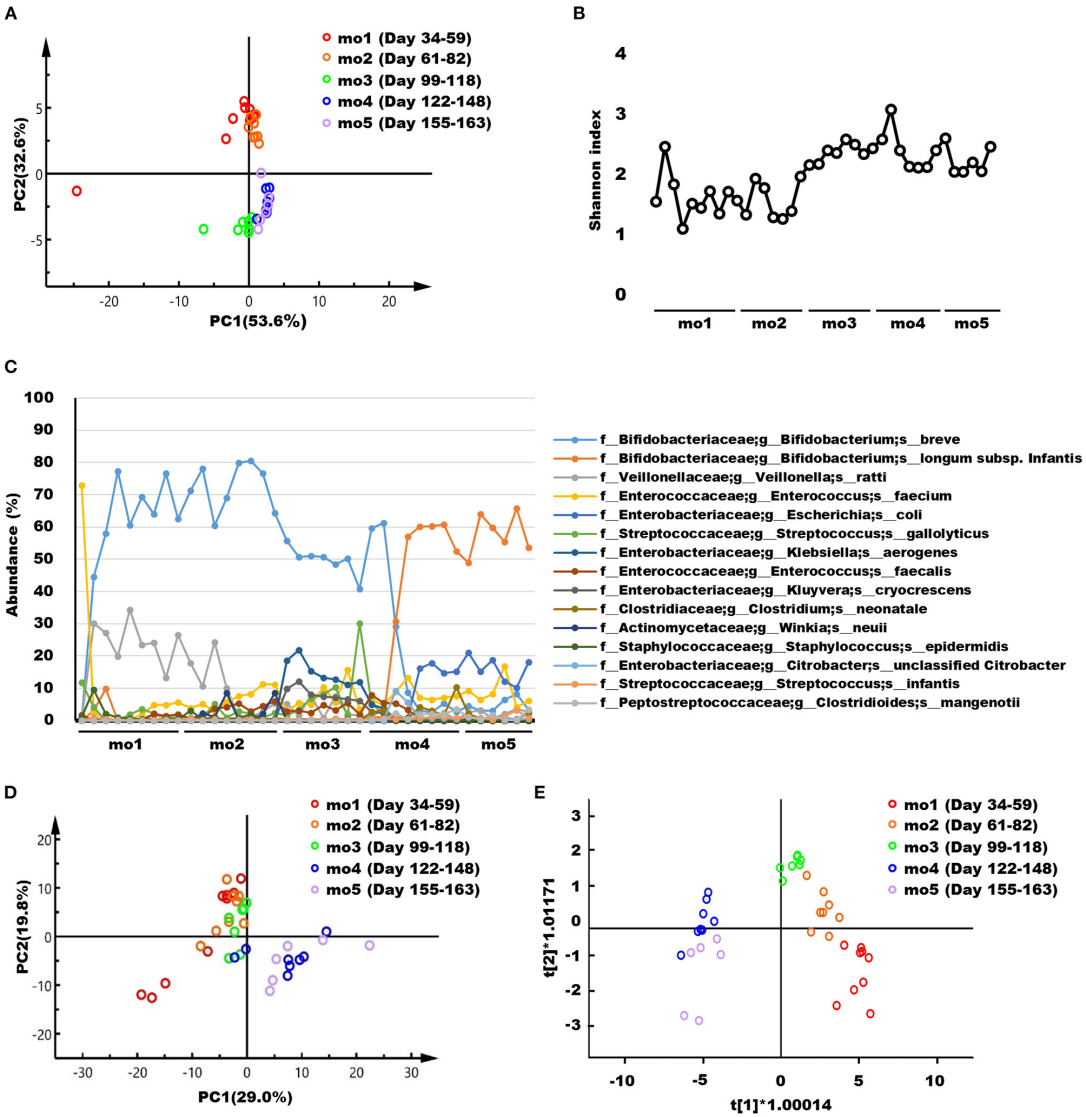
**(A)** Principal component analysis (PCA) score plots based on the weighted UniFrac distance metric of the microbiome in infant feces samples collected at months 1–5 postpartum. Each symbol represents one individual feces sample. **(B)** Changes in the microbiome α-diversity, as assessed from the Shannon index, in infant feces samples collected at months 1–5 postpartum. **(C)** Transitions of 15 bacterial species with an average abundance of 0.5% or more during the lactation period. **(D)** PCA and **(E)** Orthogonal partial least squares discriminant analysis consisting of five groups score plots derived from the NMR spectra of infant feces samples collected at months 1–5 postpartum. R2X[1] = 0.568; R2X[2] = 0.048. Each symbol represents one individual feces sample. mo, month.

The weighted UniFrac distances based on the OTUs for the microbiome in feces of the breastfed infant during each lactation period were visualized using PCA (Figure 1A) and OPLS-DA (Supplementary Figure 1). These score plots in months 1–2 and 3–5 postpartum formed clearly separate clusters, respectively. Changes in microbiome α-diversity in infant feces at each lactation point are represented by the Shannon index (Figure 1B). As the lactation period progressed, the diversity increased, especially after month 3. The transitions of 15 bacterial species with an average abundance of 0.5% or more during the lactation period are shown in Figure 1C. Furthermore, microbiome composition of breastfed infant feces at the 40 species-level at each lactation point is shown in Supplementary Table 1. In addition, quantitative PCR was performed to validate the presence of *Bifidobacterium* spp. that showed characteristic and dramatic changes in 16S RNA sequencing analysis (Supplementary Figure 2). The changes by quantitative PCR analysis of *B. breve, B. longum* subsp. *infantis*, and *B. longum* subsp. *longum* showed similar trends to those of 16S RNA sequencing analysis, which were supported by the results. Representative ^1^H NMR spectra of infant feces samples at months 1 and 5 postpartum and their enlarged views are shown in Supplementary Figure 3. Low-molecular-weight compounds, such as monosaccharides, disaccharides, oligosaccharides, SCFAs, amino acids, and their metabolites were identified, and their chemical shifts are shown in Supplementary Table 2. Changes in the feces NMR spectra during each lactation period were visualized using PCA (Figure 1D). Because the score plots for month 3 were positioned in the middle of the two clusters, the score plots derived from the NMR spectra for feces did not form clearly separate clusters. OPLS-DA (Figure 1E) performed to further compare the differences between five groups showed that the score plots derived from the NMR spectra for feces in months 1–3 and 4–5 postpartum formed separate clusters. Furthermore, the relative concentrations of infant feces metabolites in months 1–5 postpartum are shown in Supplementary Table 3. Considering the direct supply of antibacterial proteins from breast milk, the concentrations of IgA, LF, and LYZ in infant feces were measured in months 1–5 postpartum (Supplementary Table 3).

### Profiling of Components in Breast Milk

**Figure 2:**
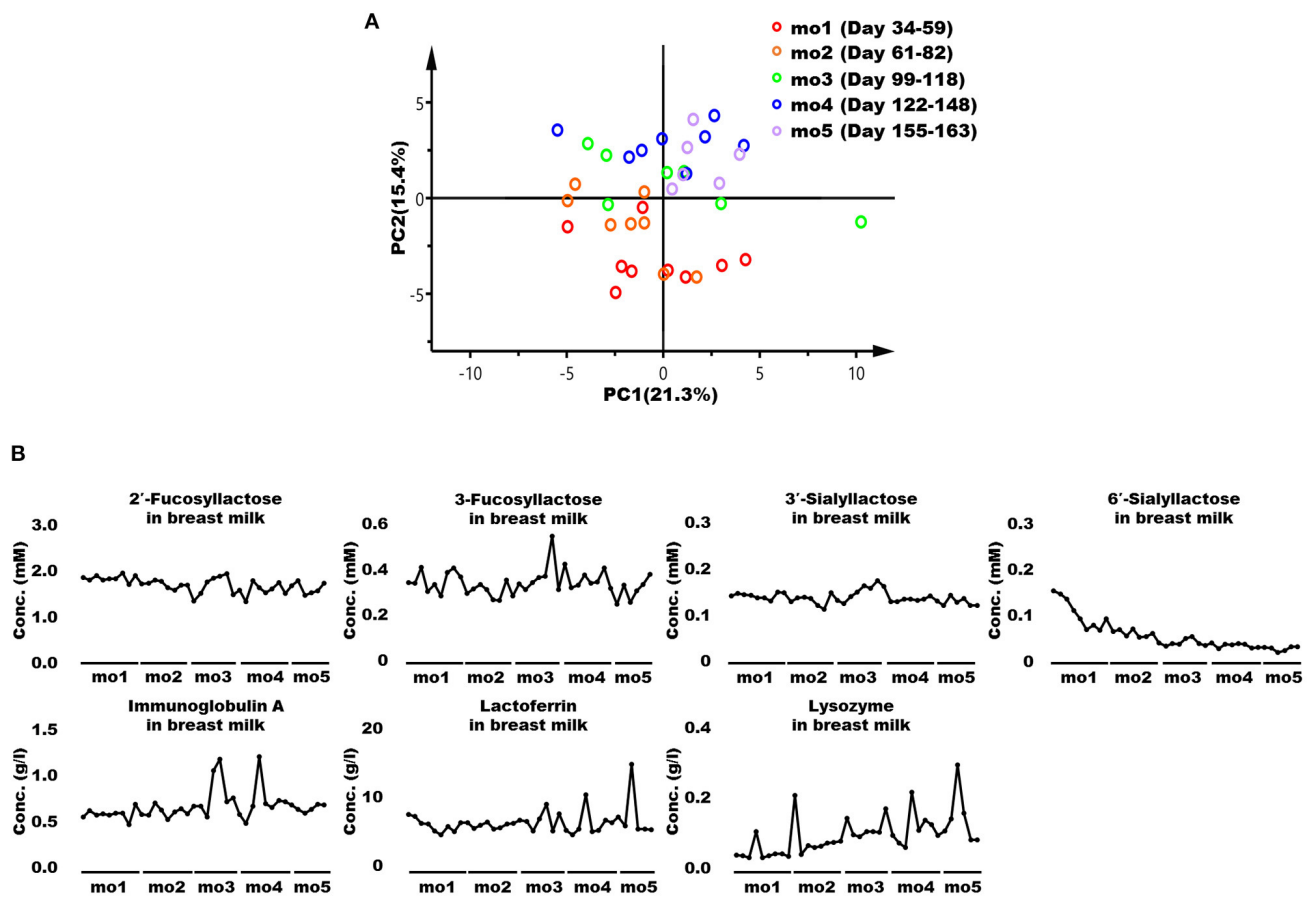
Principal component analysis (PCA) and changes in the bioactive components of breast milk. **(A)** PCA score plots derived from components such as low-molecular-weight metabolites, major nutrients, and antibacterial proteins in breast milk collected at months 1–5 postpartum. Each symbol represents an individual breast milk sample. **(B)** Changes in levels in the human milk oligosaccharides (HMOs) immunoglobulin A (IgA), lactoferrin (LF), and lysozyme (LYZ) in breast milk. mo, month.

The representative ^1^H NMR spectra of breast milk samples at months 1 and 5 postpartum and their enlarged views are shown in Supplementary Figure 4. Low-molecular-weight compounds such as monosaccharides, disaccharides, oligosaccharides, SCFAs, amino acids, and their metabolites were identified, and their chemical shifts are shown in Supplementary Table 4. Supplementary Table 5 shows the concentrations of metabolites, macronutrients (fats, proteins, carbohydrates, and total solids), and antibacterial proteins (IgA, LF, and LYZ) in breast milk at months 1–5 postpartum. Changes in breast milk components during each lactation period were visualized using PCA (Figure 2A) and OPLS-DA (Supplementary Figure 5). These score plots derived from the milk components did not form clearly separate clusters. Furthermore, Figure 2B shows fluctuations in the levels of bioactive HMOs and IgA, LF, and LYZ in breast milk. These bioactive components did not also characteristically vary throughout lactation, as shown in Figure 2B.

### Clustering Analysis of Bioactive Milk Components, the Microbiome, and Metabolites in Infant Feces

**Figure 3:**
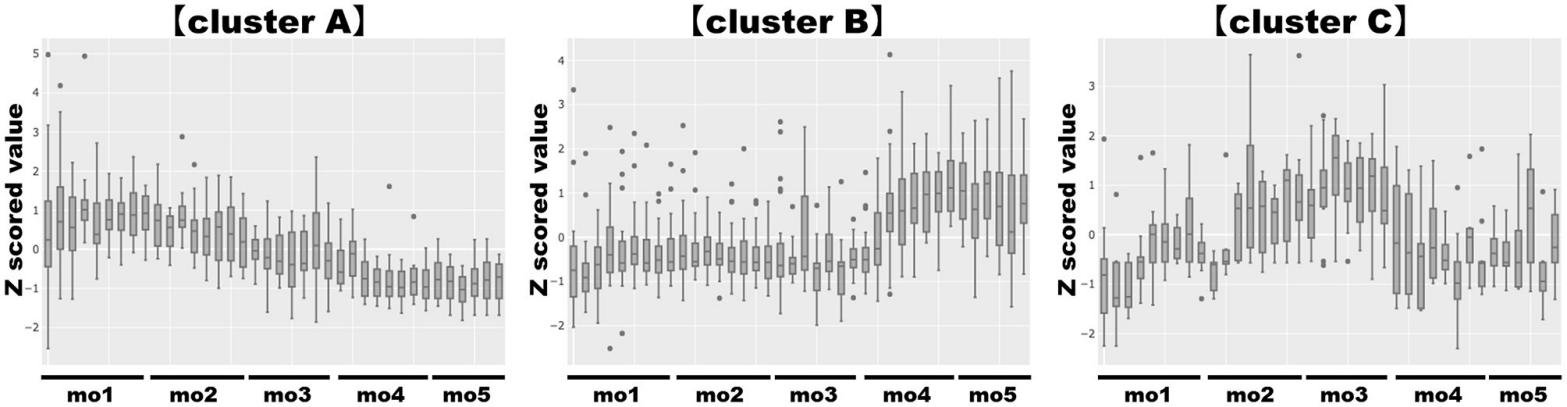
Clustering analysis of bioactive milk components, the microbiome at the species level, and the metabolites in infant feces. Those with similar characteristics (similar fluctuation patterns) were categorized into three clusters, cluster A, cluster B, and cluster C. “Cluster A” showed high values in the early-lactation period and low values in the late-lactation period; “cluster B” showed low values in the early-lactation period and high values in the late-lactation period; “cluster C” did not show a characteristic tendency throughout the lactation period. Categorized factors: (cluster A) 2′-fucosyllactose, 3-fucosyllactose, 3′-sialyllactose, 6′-sialyllactose, formate, hypoxanthine, immunoglobulin A, lactoferrin, lactose, lysozyme, trimethylamine N-oxide, tryptophan, urea, *Bifidobacterium breve, Staphylococcus epidermidis, Veillonella ratti*. (cluster B) 5-Aminopentanoate, acetate, alanine, aspartate, butyrate, cadaverine, carnitine, choline, creatine, fucose, fumarate, glucose, lactate, o-phosphocholine, propionate, pyruvate, succinate, taurine, threonine, tyrosine, *Bifidobacterium longum* subsp. *infantis, Citrobacter* unclassified *Citrobacter, Clostridioides mangenotii, Clostridium neonatale, Enterococcus faecium, Escherichia coli, Streptococcus infantis*. (cluster C) Glutamate, glycine, isoleucine, leucine, methionine, phenylalanine, valine, *Enterococcus faecalis, Klebsiella aerogenes, Kluyvera cryocrescens, Streptococcus gallolyticus, Winkia neuii*. mo, month.

**Figure 4:**
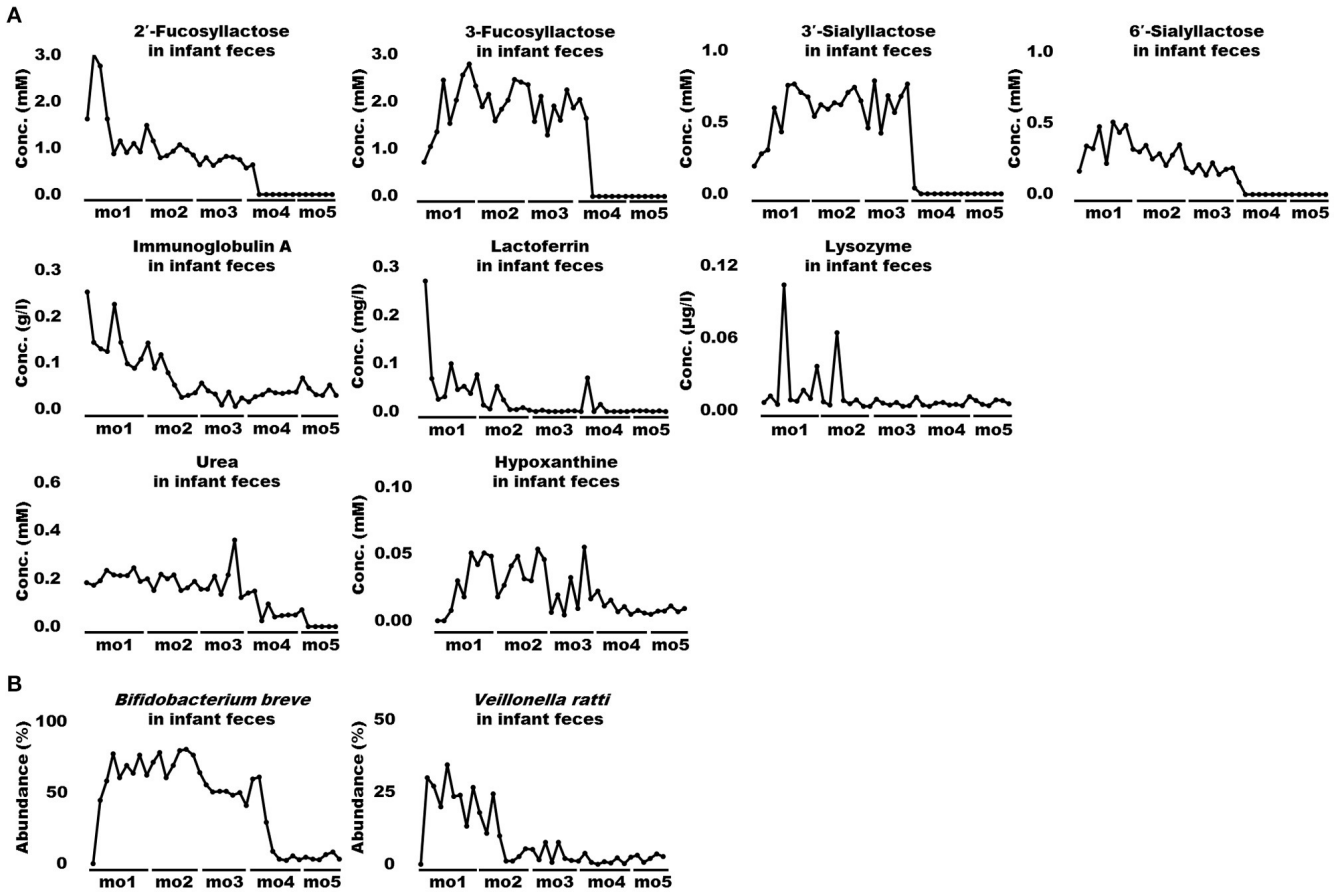
Changes in bioactive milk components, microbiome, and metabolites in infant feces with high similarity in cluster A. **(A)** Human milk oligosaccharides (2′-fucosyllactose, 3-fucosyllactose, 3′-sialyllactose, and 6′-sialyllactose), antibacterial proteins (immunoglobulin A, lactoferrin, and lysozyme), urea, and hypoxanthine in infant feces. **(B)** *Bifidobacterium breve* and *Veillonella ratti*, in infant feces. mo, month.

**Figure 5:**
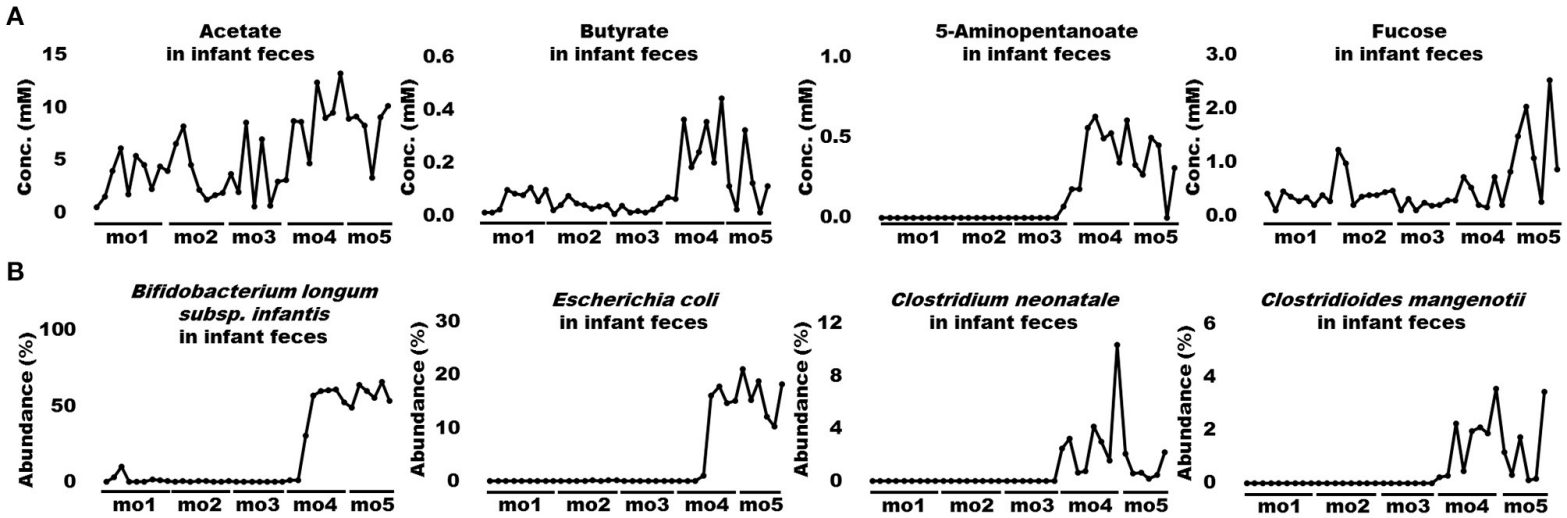
Changes in bioactive milk components, the microbiome, and metabolites in infant feces with high similarity in cluster B. **(A)** Acetate, butyrate, 5-aminopentanoate, and fucose levels in infant feces. **(B)** *Bifidobacterium longum* subsp. *infantis, Escherichia coli, Clostridium neonatale*, and *Clostridioides mangenotii* in infant feces. mo, month.

When bioactive milk components act on the infant gut microbiome via breast milk, bacterial-derived metabolites in infant feces should also fluctuate in association with bacterial changes. Therefore, association of bioactive milk components with the microbiome (at the species level; average abundance > 0.5%) and fecal metabolites of the infant was assessed with time-series clustering analysis using Python software. Three major clusters were defined, showing similar fluctuations over time (Figure 3). Values for cluster A were high and low, whereas those for cluster B were low and high during early and late-lactation, respectively, and no characteristic tendency was noted for cluster C throughout lactation. Among compounds and bacterial species classified into the same cluster, those with a particularly high similarity were visually selected. Figure 4A shows fluctuations in the levels of four HMOs, antibacterial proteins, urea, and hypoxanthine in infant feces, and Figure 4B shows transitions in the abundance of *Bifidobacterium breve* and *Veillonella ratti* in infant feces categorized into cluster A. Figure 5A shows fluctuations in the levels of acetate, butyrate, 5-aminopentanoate, and fucose in infant feces categorized into cluster B. Figure 5B shows transitions in the abundance of *Bifidobacterium longum* subsp. *infantis, E. coli, Clostridium neonatale*, and *Clostridioides mangenotii* in infant feces categorized into cluster B.

### Spearman Correlation Analysis of Bioactive Milk Components, the Infant Gut Microbiome, and Fecal Metabolites

**Figure 6:**
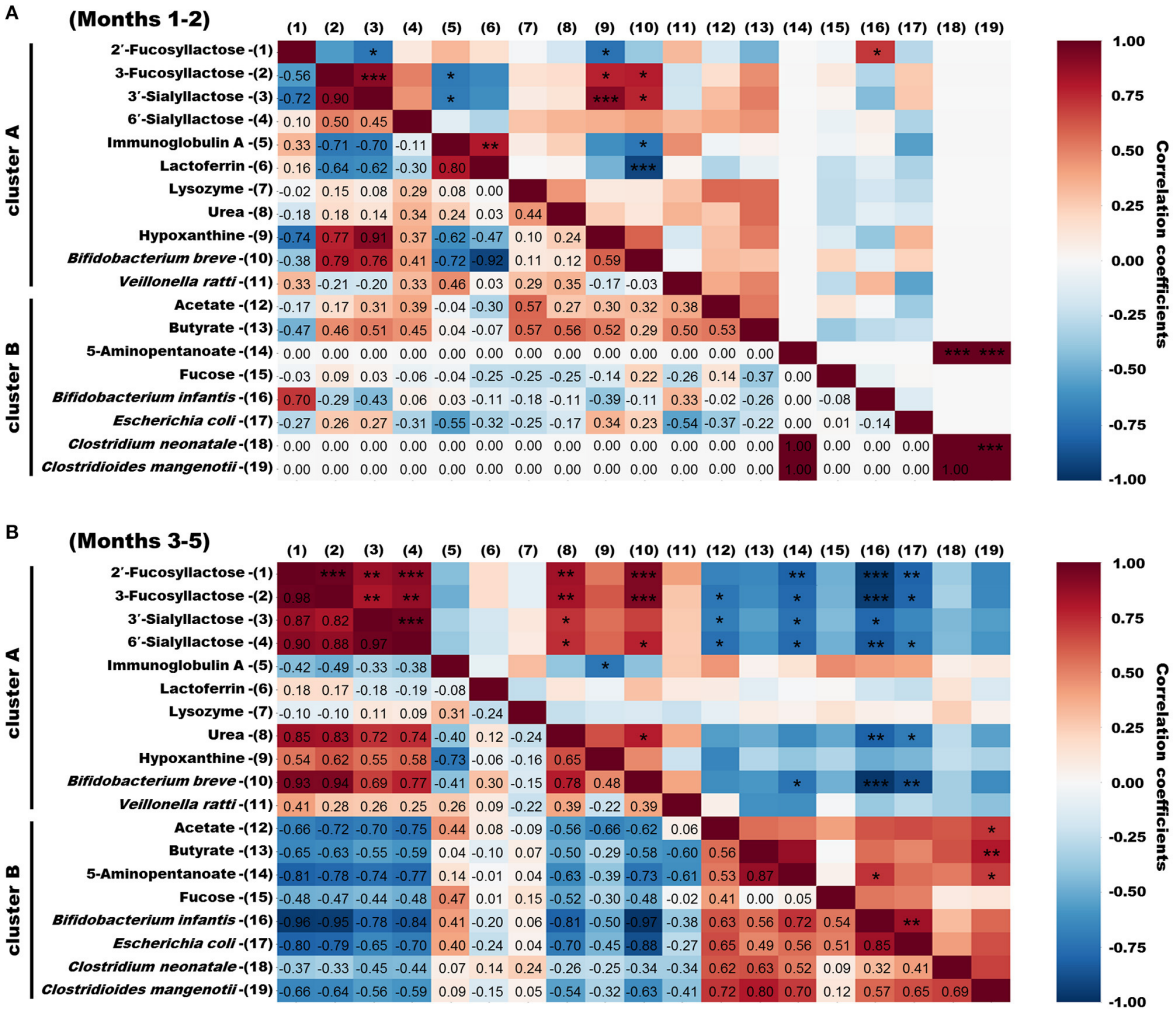
Spearman correlation heatmaps of bioactive milk components, the infant gut microbiome, and fecal metabolites in infant feces. **(A)** Spearman rank correlation coefficients of bioactive milk components, bacterial species, and metabolites in infant feces at months 1–2 postpartum with high similarity categorized into clusters A and B. **(B)** Spearman rank correlation coefficients of bioactive milk components, bacterial species, and metabolites in infant feces at months 3–5 postpartum with high similarity categorized into clusters A and B. Spearman ρ values are shown in different colors and |ρ| values are shown as *0.7–0.8, **0.8–0.9, and ^***^ >0.9. mo, month.

We used Spearman rank correlation coefficients to extract factors with significant positive or negative correlations among bioactive milk components, bacterial species, and metabolites with high similarity in clusters A and B from infant feces. The results are shown, as heatmaps, for months 1–2 and 3–5 postpartum that were clearly distinguished in microbiome of infant feces by PCA and OPLS-DA (Figures 6A,B). Few correlations were identified between bioactive milk components and the microbiome and fecal metabolites in infant feces at months 1–2 postpartum. *Bifidobacterium breve* correlated positively with 3-FL and 3′-SL (ρ = +0.79 and +0.76, respectively) and negatively correlated with IgA and LF (ρ = −0.72 and −0.92, respectively). Correlations were much closer at months 3–5 than at months 1–2. *Bifidobacterium breve* correlated significantly and negatively with *B. longum* subsp. *infantis* and *E. coli* in infant feces at months 3–5 (ρ = −0.97 and −0.88, respectively). *Bifidobacterium longum* subsp. *infantis* also significantly correlated with *E. coli* (ρ = +0.85). Correlations (ρ = +0.82 to +0.98) were significantly positive among four HMOs in infant feces. These HMOs were significantly and positively correlated (ρ = +0.69 to +0.94) with *B. breve* in cluster A in infant feces. *Bifidobacterium longum* subsp. *infantis* and *E. coli* in cluster B in infant feces significantly and negatively correlated with HMOs (ρ = −0.65 to −0.96). No bacterial species significantly correlated with the antibacterial proteins IgA, LF, and LYZ, hypoxanthine, or fucose. Acetate and butyrate levels significantly and positively correlated with *C. mangenotii* (ρ = +0.72 and +0.80, respectively), and 5-aminopentanoate levels correlated positively with *B. longum* subsp. *infantis* and *C. mangenotii* (ρ = +0.72 and +0.70, respectively). Some metabolites, such as amino acids and other bacterial species, in infant feces were classified into cluster C, which had no characteristic tendency throughout lactation.

## Discussion

Breast milk is an ideal nutritional source for infants and contains various bioactive components such as HMOs and antibacterial proteins. However, relationships between milk components and the infant gut microbiome and fecal metabolites throughout lactation remain unclear. Our PCA and OPLS-DA findings revealed different clustering profiles in the infant gut microbiome between months 1–2 and 3–5 postpartum, and the diversity of the gut microbiome also increased 3 months onwards. On the other hand, the concentrations of breast milk components throughout lactation showed some variation but did not show a characteristic tendency. This indicated that the gut microbiome of the breastfed infant fluctuated after month 3 postpartum, even though milk components did not change. These changes in the infant intestinal tract might translate into optimization for the efficient digestion and absorption of breast milk. In fact, it has been reported that after the 3rd month of life, the functional maturation of the gastrointestinal tract progresses rapidly and the fecal properties change dramatically (30). In the OPLS-DA following PCA of the infant gut microbiome and fecal metabolites, score plots of the microbiome formed clusters in months 1–2 and 3–5 postpartum, whereas score plots of the fecal metabolites formed clusters in months 1–3 and 4–5 postpartum. The difference in cluster formation in the postnatal period suggested that the gut microbiome changed first, followed by its fecal metabolites. From a comprehensive point of view, the infant gut microbiota might first respond to changes in the infant's body with gastrointestinal maturation, followed by changes in fecal metabolites.

Interestingly, the concentrations of HMOs such as 2′-FL, 3-FL, 3′-SL, and 6′-SL in breast milk were almost constant, whereas in infant feces these HMOs were no longer detected after the beginning of month 4. Firstly, this change in infant feces suggests that the amounts of HMOs delivered into infant feces via breast milk changed between early and late lactation. The structural characteristics and prebiotic effects of HMOs assimilated by intestinal bacteria protect against pathogens (31). The effects of HMOs might change as infants grow. Secondly, the disappearance of HMOs in infant feces suggest that the infant's gut microbiome began to metabolize these HMOs from month 4 onwards. Importantly, Spearman analysis of infant feces at months 3–5 postpartum revealed significant positive correlations between HMOs and *B. breve*. In contrast, HMOs and *B. longum* subsp. *infantis* were significantly and negatively correlated. Spearman analysis also revealed a significantly negative correlation between *B. breve* and *B. longum* subsp. *infantis* in infant feces in 3–5 months. Several *in vitro* studies have reported that 2′-FL, 3-FL, 3′-SL, and 6′-SL are not metabolized by *B. breve* but are metabolized by *B. longum* subsp. *infantis* due to the difference in the HMO capitalization mechanism. The fucosidase belonging to GH29, which hydrolyzes α-1,3-linked fucosyl lactose, is not present in most of *B. breve*, but only in *B. longum* subsp. *infantis* (32). Besides, *B. breve* and *B. longum* subsp. *infantis* have different structures of ATP-binding cassette transporter of FL with high frequency (33). These differences in enzyme and transporter might also be responsible for the rapid FL utilization of *B. longum* subsp. *infantis* after the beginning of month 4. Besides, it is possible that sialidases which were detected commonly in *B. longum* subsp. *infantis* and *B. bifidum* affect 3′-SL and 6′-SL utilization (34). Given the differences in these multiple HMO capitalization mechanisms, it is inferred that the dramatic decrease in HMOs in the infant feces was probably because of the “species switching” from *B. breve* to *B. longum* subsp. *infantis*. Notably, some strains of *B. breve* are known to metabolize 2′-FL and 3-FL (32). The *B. breve* detected in the infant feces of this study could have been a strain that does not metabolize 2′-FL and 3-FL. *Enterococcus faecalis*, categorized in cluster C, which did not exhibit a characteristic tendency throughout lactation, remained abundant during the switch from *B. breve* to *B. longum* subsp. *infantis*. Exopolysaccharides produced by *E. faecalis* promote the growth of *B. longum* rather than *B. breve* (35). Thus, the growth of *B. longum* could explain the switch from *B. breve* to *B. longum* subsp. *infantis* found herein. Furthermore, *E. faecalis* modulates inflammation in the gastrointestinal tract during the first few years of life and affects the development of intestinal immunity associated with allergies (36, 37). These facts indicate that the increase in *E. faecalis* at 2–4 months indirectly and directly contributed to the development of the gastrointestinal tract of the infant studied herein. *Escherichia coli* was relatively significantly and negatively correlated with HMOs in infant feces. Fucosylated and sialylated glycans in breast milk can bind to *E. coli* and suppress infection (38, 39). Therefore, *E. coli* abundance in infant feces might have increased due to the depletion of HMOs. *Escherichia coli* produces acetate via acid fermentation under anaerobic conditions (40). Thus, elevated acetate levels in the infant feces might have been partly due to *E, coli*, even though the correlation coefficient between acetate and *E. coli* was <0.70 at months 3–5 postpartum. Furthermore, supplementing preterm infants with bifidobacteria increases fecal acetate levels via metabolized HMOs and consequently promotes the defense of epithelial and mucosal dendritic cells (41). The genus *Bifidobacterium* has a specific glycolytic system called the “bifid shunt” that efficiently produces lactate and acetate from glucose, a component of HMOs. Thus, the metabolism of HMOs by *B. longum* subsp. *infantis* might have partly contributed to the elevated acetate in infant feces later during lactation in the present study. Several previous studies have reported that the abundance of *B. longum* subsp. *infantis* and *E. coli* are negatively correlated in infants within the first month postpartum or preterm infants (42, 43), contrary to our results. These differences in correlation might be due to the different characteristics of the subjects participated in previous studies and our study. In future clinical trials, these relationships might become apparent by categorizing infants based on the presence ratio of *Bifidobacterium*/*Enterobacteriaceae* in the gut microbiome and following mother-infant pairs individually. Fecal butyrate significantly and positively correlated with *C. mangenotii* at 3–5 months. The results of PICRUSt analysis and an anaerobic culture system have shown that *C. mangenotii* is a potential butyrate producer (44). Therefore, the elevated butyrate levels in the infant feces are thought to be a result of increased *C. mangenotii* abundance during late lactation. Butyrate in the intestinal tract confers benefits, such as anti-inflammatory effects on epithelial cells, to hosts (45). Moreover, it contributes to maturation of the immune system by modulating the differentiation of regulatory T cells in the large intestine (46). However, there are some evidence that differentiation of regulatory T cells in adaptive immune programming is critical within 100 days of age, so the elevated butyrate in the late-lactation period might not be directly involved (47, 48). On the other hand, butyrate also has direct immune-activating functions, such as inhibiting the adhesion of pathogenic bacteria or improving the gastrointestinal barrier (49). Therefore, elevated butyrate levels during late lactation could help immune modulation in infants indirectly and directly. Breast milk contains acetate and butyrate, but they are rapidly absorbed in the upper gastrointestinal tract (50, 51). These metabolites in infant feces are probably produced via fermentation by the gut microbiome.

The changes of antibacterial proteins IgA, LF, and LYZ in breast milk were not characteristic, but those in infant feces were categorized into cluster A, and tended to be higher in month 1–2. These results suggest that antibacterial proteins in breast milk are not necessarily reflected by the content of the influx into the digestive tract of the infant. Secretory IgA is stable against proteolytic enzymes in infant's gastrointestinal tract, and there binds to bacterial and viral antigens, promoting inhibition of attachment to the mucosal lining (17). The presence of high levels of IgA in months1–2 in our study might contribute to the prevention of infection in the gastrointestinal tract of infants with immature mucosal defenses. Although LF and LYZ have their own anti-infection properties, digested forms exert stronger antibacterial activities than the full-length intact forms (52, 53). The disappearance of the LF and LYZ since month 3 in infant feces might mean that the digestion of these intact proteins started at month 3 postpartum, as the digestive capacity of the infant developed. Furthermore, LF in infant feces significantly and negatively correlated with *B. breve* at months 1–2 postpartum. Some peptides isolated from human milk exert strong bifidogenic effects on *B. bifidum, B. breve*, and *B. longum* and are resistant to digestive enzymes (54). Thus, digested LF in the gastrointestinal tract might promote the growth of *B. breve* at months 1–2 despite the immature digestive function of the infant. At months 3–5 postpartum, antibacterial proteins (IgA, LF, LYZ) in infant feces did not significantly correlate with any bacterial species detected in the present study. However, antibacterial proteins are supplied via breast milk to the digestive tract, where they modify the infant gut microbiome (55, 56). Further studies are needed to clarify the effects of antibacterial proteins in breast milk on the infant gut microbiome.

Urea and hypoxanthine are metabolites that are respectively generated by amino and nucleic acid metabolism; these were categorized into cluster A, which was respectively abundant and scant during early and late lactation. However, as the amounts of amino acids (or proteins) and nucleic acids required for a developing infant significantly change during lactation, the relationship between the gut microbiome and these nutritional components remains controversial (57). The genus Clostridium produces 5-aminopentanoate from proline (58). Therefore, the increase in 5-aminopentanoate found herein might be due to an increase in the abundance of *C. neonatale* despite the absence of a significant correlation with 5-aminopentanoate. As far as we can ascertain, the function of 5-aminopentanoate in infants is unknown. However, 5-aminopentanoate can be a biomarker of cerebral ischemia (59), and it might therefore play an important role in the development of brain function in infants. Spearman analysis did not identify significant correlations between fecal fucose and any factors. Since fucose is a component of fucosylated HMOs (16), it might have been produced in infant feces via the metabolism of 2′-FL and 3-FL. However, the physiological significance of fucose requires further investigation. In addition, no characteristic fluctuations were shown for metabolites and macronutrients such as fat, protein, and carbohydrate in breast milk in the current study. Since macronutrients have been reported to be directly associated with the gut microbiome of the infant and the modulation of the immune system (4), further investigation of the nutrients supplied via breast milk is warranted.

The major limitation of this pilot study is that we used a model of only one mother–infant pair. However, this is the first study to examine dynamic associations of breast milk with breastfed infant feces on a weekly basis over the entire lactation period, which have only been studied monthly previously. Frequent sampling data monthly may be used as reference for future large-scale mother–infant studies. For example, given the possibility of dramatic changes in the gut microbiome of other infants during month 4 postpartum, it might be suggested that frequent sampling is necessary during this period. Although it is challenging to collect samples weekly in a clinical study, the dynamic associations between breast milk and infant feces determined in this study will need to be confirmed using a larger sample size. Further, we did not include colostrum and transitional milk secreted during the month 1 postpartum in our analysis (60). This exclusion might explain the limited fluctuations in breast milk components throughout lactation in the present study. However, colostrum and transitional milk contain higher amounts of functional components, such as antimicrobial proteins and immune modulators, than mature milk (61). The consumption of colostrum and transitional milk within month 1 postpartum may have a long-term effect on infant gut microbiome and fecal metabolites. Another limitation is that the involvement of HMOs such as LNT and LNFP I was not investigated. Indeed, these HMOs may be present in breast milk and infant feces, although they could not be detected because of the overlap of NMR spectra derived from compounds with close chemical shift values. Most of the genus *Bifidobacterium* has metabolic pathways and enzymes to utilize LNT and LNFP I, and can capitalize on these HMOs (62). The utilization of these HMOs by the gut microbiome might be involved in the “species switching” from *B. breve* to *B. longum* subsp. *infantis*. Furthemore, these HMOs are also known to be involved in the development and maturation of infants and may explain the fluctuating gut microbiome observed in this study. Therefore, further studies will be needed in the future to confirm the role of these HMOs (63). Finally, another limitation of this study is that the amount of breast milk consumed by the infant was not measured. If the breastfeeding amount were accurately measured, it would be possible to estimate how much HMOs in breast milk are supplied to the infant and utilized in the infant's gut microbiome. In future clinical trials, data on the amount of breast feeding will be needed to verify in detail the dramatic changes in HMOs observed in this study.

In conclusion, microbiome analysis, NMR metabolomics, and Python's clustering and correlation analyses showed that the gut microbiome and fecal metabolites of the infant were dynamically associated with bioactive milk components during a certain lactation period. In particular, HMOs in the breast milk did not fluctuate throughout the lactation period but disappeared from the infant feces after the 4th lactation month. Moreover, at the same time species switching from *B. breve* to *B. longum* subsp. *infantis, E. coli*, and *C. mangenotii* occurred, accompanied by an increase in the levels of metabolites, such as acetate and butyrate, in the infant feces. Importantly, our data suggest that the changes in metabolites in the infant feces might be linked to benefits such as the maturation of immune function and protection against infection at a certain period during lactation. Therefore, bioactive milk components, such as HMOs, might play different roles in the exclusively breastfed infants depending on the lactation period. In particular, the increase in metabolites such as acetate and butyrate in the late-lactation period might reflect the switch from developmental benefits to the infant via breastfeeding to benefits conferred through metabolism in the infant or its own gut microbiome. It has also been reported that the microbiome in breast milk, and bacteria on the mother's skin and the infant's oral cavity, might contribute to changes in the infant gut microbiome (64). To develop a mechanistic understanding of the complex interactions among microbial species, more detailed studies are required, such as co-culturing the bacterial species that fluctuated in this study. However, the present findings could contribute to understanding the relationship between bioactive milk components and the infant gut microbiome and to the development of supplementary nutritional foods for breastfed infants during lactation.

## Data Availability Statement

The datasets presented in this study can be found in online repositories. The names of the repository/repositories and accession number(s) can be found at: DDBJ; Accession PRJDB12676.

## Ethics Statement

The studies involving human participants were reviewed and approved by the Japan Conference of Clinical Research (Approval Number: BONYU-01). The patients/participants provided their written informed consent to participate in this study.

## Author Contributions

YK, HI, YT, SN, and TA designed the study. YK, DK, YO, and SN analyzed the data and interpreted the result. NS contributed to discussions including the experimental schedule. YK conducted the experiments and wrote the paper. YK is primarily responsible for the final content. All authors have read and approved the final manuscript.

## Funding

This research was funded by Morinaga Milk Industry Co., Ltd.

## Conflict of Interest

YK, NS, HI, and YT are employees of Morinaga Milk Industry Co., Ltd. The remaining authors declare that the research was conducted in the absence of any commercial or financial relationships that could be construed as a potential conflict of interest. The authors declare that this study was funded by Morinaga Milk Industry Co., Ltd. The funder was not involved in the study design, collection, analysis, interpretation of data, the writing of this article, or the decision to submit it for publication.

## Publisher's Note

All claims expressed in this article are solely those of the authors and do not necessarily represent those of their affiliated organizations, or those of the publisher, the editors and the reviewers. Any product that may be evaluated in this article, or claim that may be made by its manufacturer, is not guaranteed or endorsed by the publisher.

## Abbreviations

FL: Fucosyllactose
HMO: Human milk oligosaccharide
Ig: Immunoglobulin
LF: Lactoferrin
LNFP: Lacto-*N*-fucopentaose
LNT: Lacto-*N*-tetraose
LYZ: Lysozyme
OPLS-DA: Orthogonal partial least squares discriminant analysis
OTUs: Operational taxonomic units
PC: Principal components
PCA: Principal component analysis
SCFA: Short-chain fatty acids
SL: Sialyllactose
TSP: 3-(Trimethylsilyl)propionic acid-d4 sodium salt.
