# Supplementary material for: Dynamic Associations of Milk Components With the Infant Gut Microbiome and Fecal Metabolites in a Mother–Infant Model by Microbiome, NMR Metabolomic, and Time-Series Clustering Analyses
Source: Front Nutr. 2022 Jan 7;8:813690. doi: 10.3389/fnut.2021.813690 (PMC8780135; doi:10.3389/fnut.2021.813690)
Supplement: Supplementary file 1 [file Data_Sheet_1.PDF]

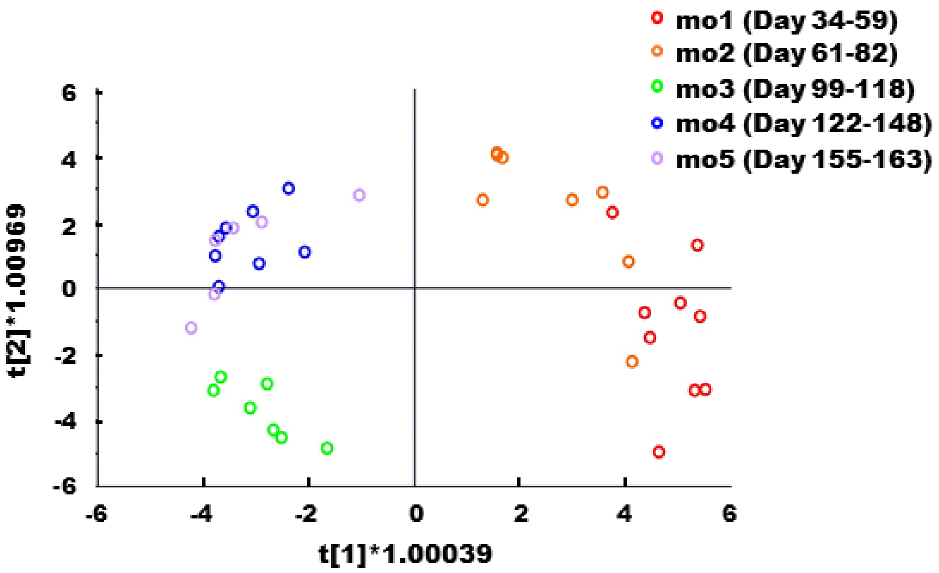

Supplementary Figure 1. Orthogonal partial least squares discriminant analysis (OPLS-DA) score plots of the microbiome in infant feces samples.

OPLS-DA consisting of five groups score plots based on the weighted UniFrac distance metric of the microbiome in infant feces samples collected at months 1–5 postpartum. Each symbol represents one individual feces sample. $R^2X[1] = 0.351$; $R^2X[2] = 0.194$. mo, month.

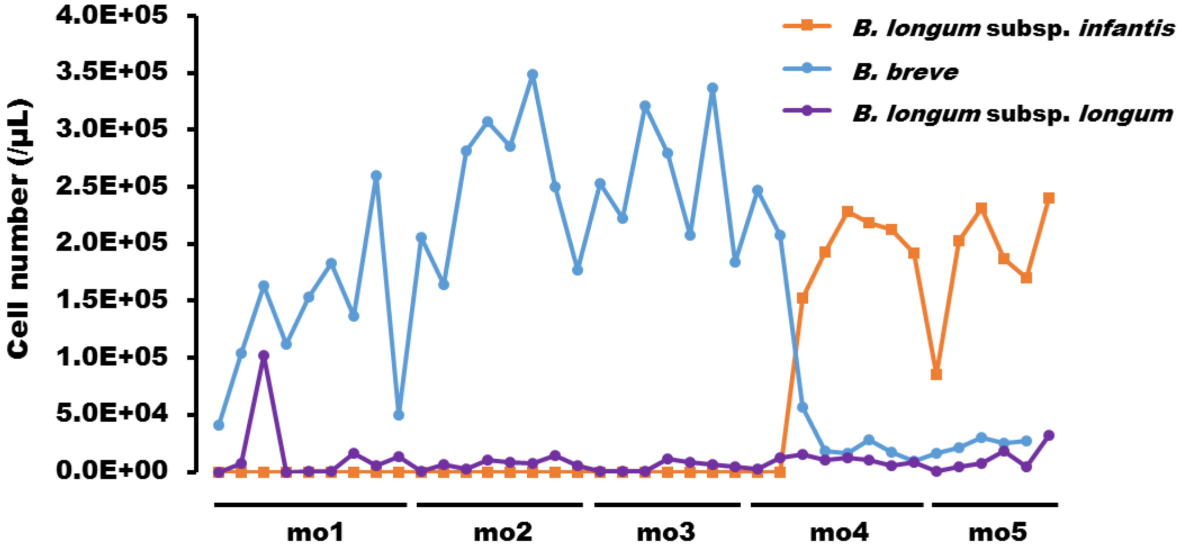

Supplementary Figure 2. Cell numbers of Bifidobacterium species in infant feces at months 1–5 postpartum.

Cell numbers of *B. longum* subsp. *infantis*, *B. breve*, and *B. longum* subsp. *longum* in infant feces during the lactation period analysed by real-time PCR method. mo, month.

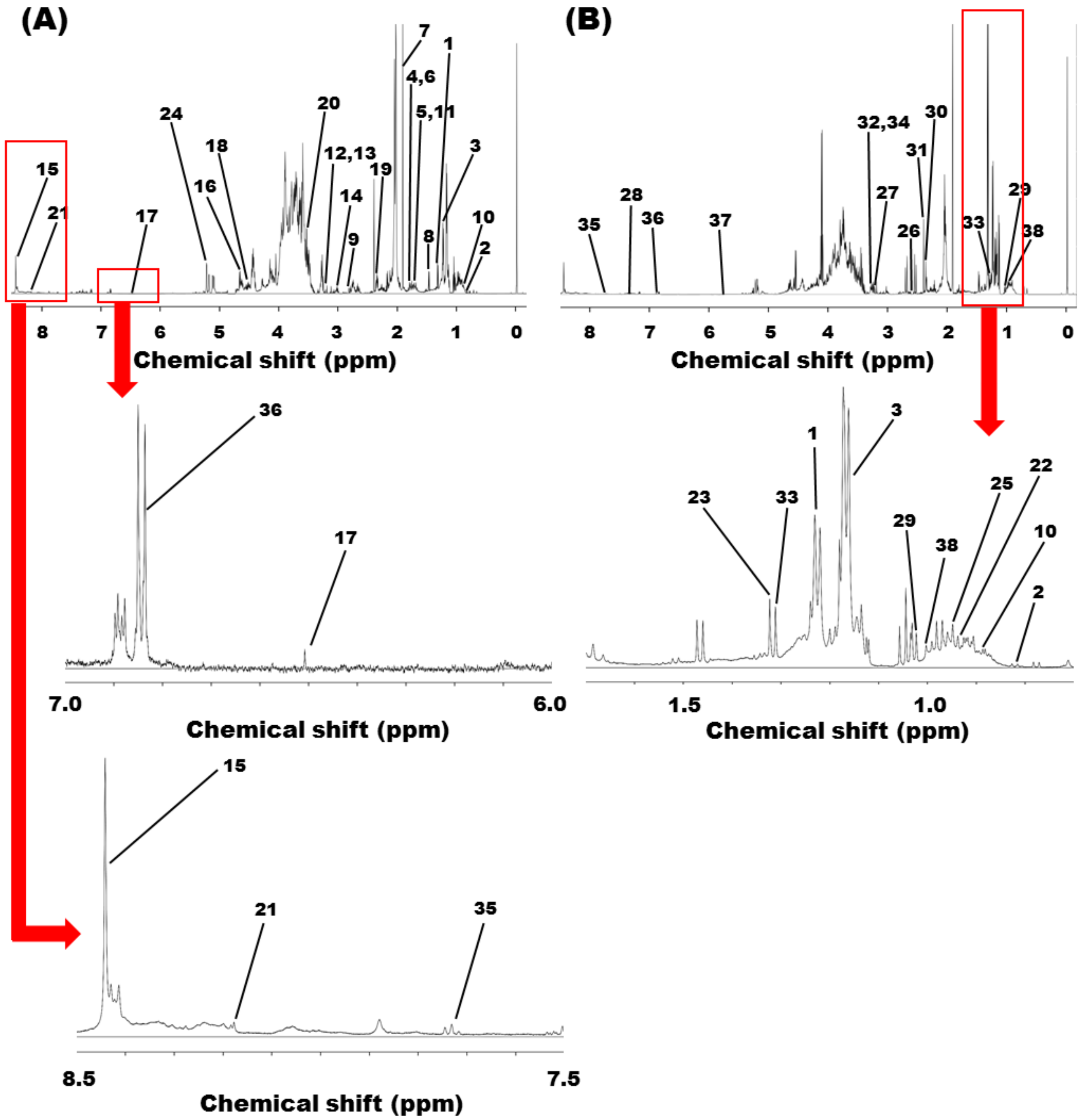

Supplementary Figure 3. ^{1}H NMR spectra of infant feces samples at months 1 and 5 postpartum. Typical 600 MHz ^{1}H NMR spectra of infant faeces samples at months (**A**) 1 and (**B**) 5. Identified metabolites: 1: 2'-fucosyllactose; 2: 2-hydroxyisovalerate; 3: 3-fucosyllactose; 4: 3'-sialyllactose; 5: 5-aminopentanoate; 6: 6'-sialyllactose; 7: acetate; 8: alanine; 9: aspartate; 10: butyrate; 11: cadaverine; 12: carnitine; 13: choline; 14: creatine; 15: formate; 16: fucose; 17: fumarate; 18: glucose; 19: glutamate; 20: glycine; 21: hypoxanthine; 22: isoleucine; 23: lactate; 24: lactose; 25: leucine; 26: methionine; 27: o-phosphocholine; 28: phenylalanine; 29: propionate; 30: pyruvate; 31: succinate; 32: taurine; 33: threonine; 34: trimethylamine N-oxide; 35: tryptophan; 36: tyrosine; 37: urea; 38: valine.

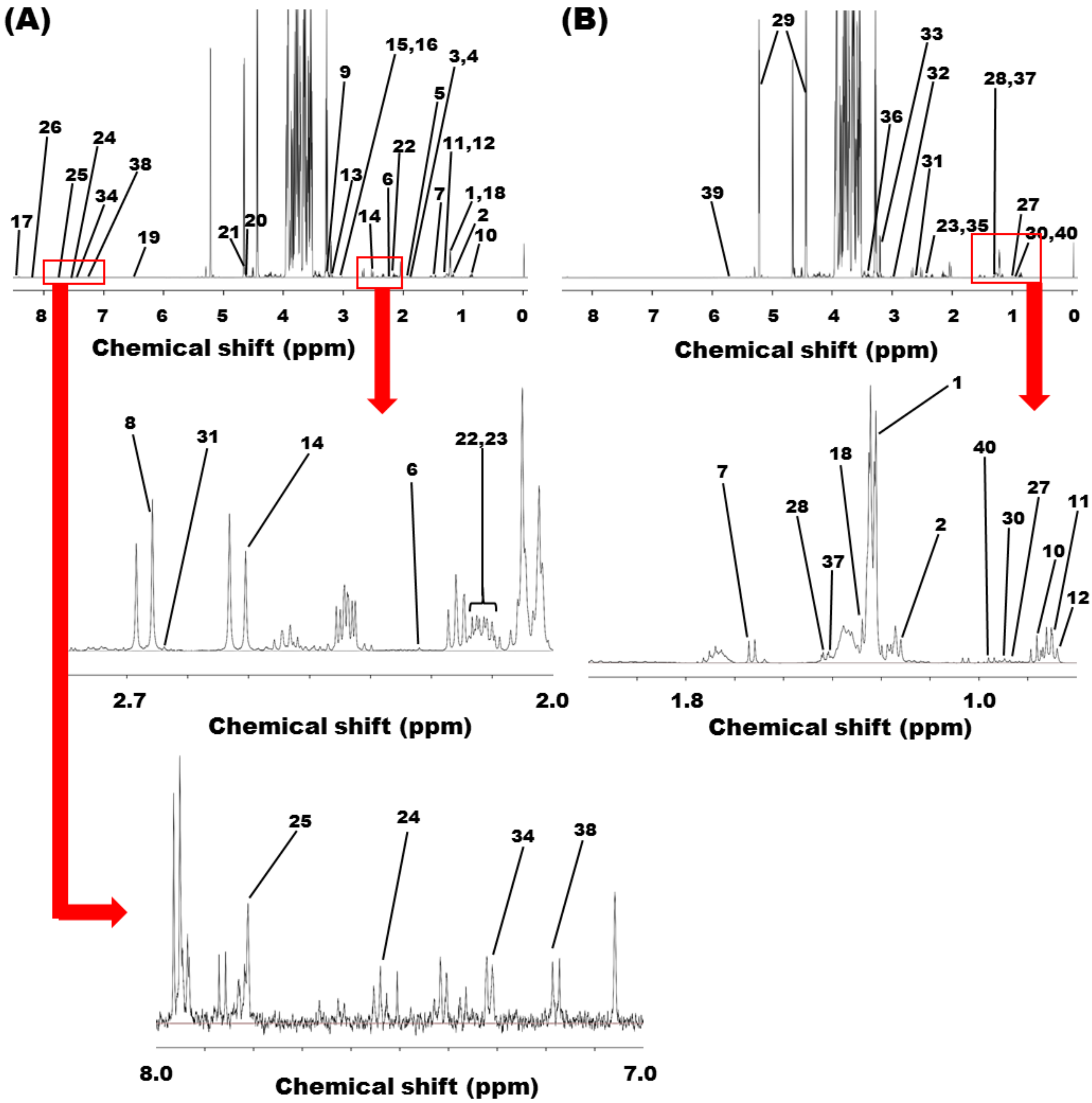

Supplementary Figure 4. ^{1}H NMR spectra of breast milk samples at months 1 and 5 postpartum. Typical 600 MHz ^{1}H NMR spectra of breast milk samples at months (**A**) 1 and (**B**) 5. Identified metabolites: 1: 2′-fucosyllactose; 2: 3-fucosyllactose; 3: 3′-sialyllactose; 4: 6′-sialyllactose; 5: acetate; 6: acetone; 7: alanine; 8: aspartate; 9: betaine; 10: butyrate; 11: caprate; 12: caprylate; 13: choline; 14: citrate; 15: creatine; 16: creatinine; 17: formate; 18: fucose; 19: fumarate; 20: galactose; 21: glucose; 22: glutamate; 23: glutamine; 24: hippurate; 25: histidine; 26: hypoxanthine; 27: isoleucine; 28: lactate; 29: lactose; 30: leucine; 31: methionine; 32: 2-oxoglutarate; 33: o-phosphocholine; 34: phenylalanine; 35: succinate; 36: taurine; 37: threonine; 38: tyrosine; 39: urea; 40: valine.

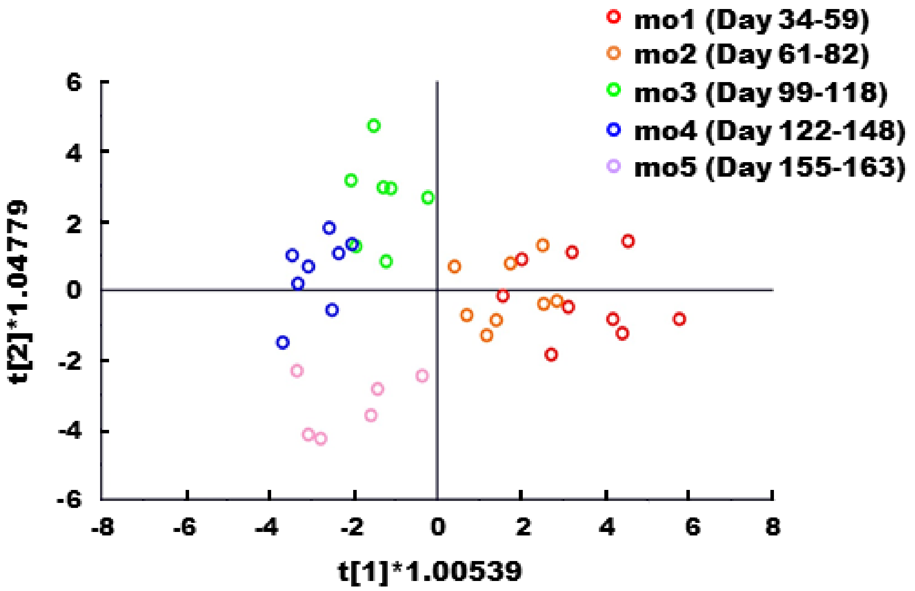

Supplementary Figure 5 Orthogonal partial least squares discriminant analysis (OPLS-DA) score plots in breast milk components.

OPLS-DA consisting of five groups score plots derived from components such as low-molecular-weight metabolites, major nutrients, and antibacterial proteins in breast milk collected at months 1–5 postpartum. Each symbol represents one individual milk sample. $R^2X[1] = 0.154$; $R^2X[2] = 0.088$. mo, month.

Supplementary Table 1. Relative abundance of the microbial species in infant feces samples collected at months 1–5 postpartum

	Microbial species	Month 1									Month 2				
		Day 34	Day 36	Day 41	Day 46	Day 51	Day 53	Day 58-1	Day 58-2	Day 59	Day 61	Day 62	Day 66	Day 69	Day 72
						%							%		
1	f__Bifidobacteriaceae;g__Bifidobacterium;s__longum subsp. Infantis	0.00	3.13	9.94	0.00	0.06	0.00	1.73	1.02	0.89	0.21	0.60	0.15	0.74	0.61
2	f__Bifidobacteriaceae;g__Bifidobacterium;s__breve	0.13	44.61	58.09	77.39	60.69	69.31	63.97	76.65	62.53	71.32	78.16	60.51	69.04	79.87
3	f__Bifidobacteriaceae;g__Bifidobacterium;s__pseudocatenulatum	0.00	3.34	0.50	0.00	0.00	0.00	0.00	0.00	0.00	0.00	0.00	0.00	0.00	0.00
4	f__Actinomycetaceae;g__Winkia;s__neuii	0.00	0.00	0.00	0.00	0.00	0.00	0.16	0.00	0.32	0.12	2.21	1.18	8.64	0.23
5	f__Enterobacteriaceae;g__Escherichia;s__coli	0.00	0.00	0.00	0.00	0.00	0.00	0.00	0.00	0.00	0.00	0.00	0.00	0.00	0.09
6	f__Enterobacteriaceae;g__Klebsiella;s__aerogenes	0.00	0.00	0.00	0.00	0.00	0.00	0.00	0.00	0.00	0.00	0.00	0.00	0.00	0.00
7	f__Enterobacteriaceae;g__Kluyvera;s__cryocrescens	0.00	0.00	0.00	0.00	0.00	0.00	0.00	0.00	0.00	0.00	0.00	0.00	0.00	0.00
8	f__Enterobacteriaceae;g__Citrobacter;s__unclassified Citrobacter	0.00	0.00	0.00	0.00	0.00	0.00	0.00	0.00	0.00	0.00	0.00	0.00	0.00	0.00
9	f__Enterobacteriaceae;g__Citrobacter;s__farmeri	0.00	0.00	0.00	0.00	0.00	0.00	0.00	0.00	0.00	0.00	0.00	0.00	0.00	0.00
10	f__Enterobacteriaceae;g__Raoultella;s__terrigena	0.00	0.00	0.00	0.00	0.00	0.00	0.00	0.00	0.00	0.00	0.00	0.00	0.00	0.00
11	f__Enterobacteriaceae;g__Citrobacter;s__freundii	0.00	0.00	0.00	0.00	0.00	0.00	0.00	0.00	0.00	0.00	0.00	0.00	0.00	0.00
12	f__Enterobacteriaceae;g__Enterobacter;s__hormaechei	0.00	0.00	0.00	0.00	0.00	0.00	0.00	0.00	0.00	0.00	0.00	0.00	0.00	0.00
13	f__Enterobacteriaceae;g__Citrobacter;s__koseri	0.00	0.00	0.00	0.00	0.00	0.00	0.00	0.00	0.00	0.00	0.00	0.00	0.00	0.00
14	f__Pasteurellaceae;g__Haemophilus;s__parainfluenzae	10.28	0.00	0.00	0.00	0.00	0.00	0.00	0.00	0.00	0.00	0.00	0.00	0.00	0.00
15	f__Streptococcaceae;g__Streptococcus;s__gallolyticus	11.78	4.15	0.00	0.00	1.85	3.48	2.18	1.02	1.00	0.96	1.01	5.17	1.01	2.87
16	f__Streptococcaceae;g__Streptococcus;s__infantis	0.00	0.92	0.40	0.38	0.19	0.13	0.19	0.35	0.08	0.85	0.20	0.46	0.00	0.29
17	f__Streptococcaceae;g__Streptococcus;s__salivarius	0.38	1.20	0.89	0.99	1.48	0.64	0.30	0.35	0.49	0.75	0.37	0.33	0.20	0.60
18	f__Streptococcaceae;g__Streptococcus;s__peroris	0.00	0.00	0.00	0.00	0.00	0.00	0.00	0.00	0.00	0.00	0.00	0.00	0.00	0.00
19	f__Enterococcaceae;g__Enterococcus;s__faecium	72.90	2.43	0.70	0.13	0.44	1.98	4.89	4.91	5.66	3.79	5.19	2.88	5.80	7.56
20	f__Enterococcaceae;g__Enterococcus;s__faecalis	1.79	0.00	0.00	0.00	0.00	0.31	2.09	1.40	1.95	0.65	1.05	3.64	4.24	5.28
21	f__Enterococcaceae;g__Enterococcus;s__casseliflavus	0.00	0.00	0.00	0.00	0.00	0.00	0.00	0.00	0.00	0.00	0.00	0.00	0.00	0.00
22	f__Streptococcaceae;g__Streptococcus;s__thermophilus	0.00	0.00	0.00	0.00	0.00	0.00	0.00	0.00	0.00	0.00	0.00	0.00	0.00	0.00
23	f__Streptococcaceae;g__Streptococcus;s__sp.	0.00	0.00	0.00	0.00	0.00	0.00	0.00	0.00	0.00	0.00	0.00	0.00	0.00	0.00
24	f__Staphylococcaceae;g__Staphylococcus;s__epidermidis	1.12	9.56	2.14	1.02	0.67	0.48	0.17	0.64	0.24	3.03	0.41	0.90	0.20	0.74
25	f__Staphylococcaceae;g__Staphylococcus;s__aureus	0.00	0.00	0.00	0.00	0.00	0.00	0.00	0.00	0.00	0.00	0.00	0.00	0.00	0.00
26	f__Staphylococcaceae;g__Staphylococcus;s__lugdunensis	0.14	0.30	0.00	0.00	0.00	0.15	0.20	0.39	0.20	0.37	0.00	0.23	0.14	0.42
27	f__Paenibacillaceae;g__Paenibacillus;s__sp.	0.00	0.00	0.00	0.00	0.00	0.00	0.00	0.00	0.00	0.00	0.00	0.00	0.00	0.00
28	f__Bacillales Family XI. Incertae Sedis;g__Gemella;s__haemolysans	0.00	0.28	0.19	0.17	0.22	0.10	0.06	0.00	0.00	0.09	0.12	0.08	0.00	0.19
29	f__Clostridiaceae;g__Hungatella;s__effluvii	0.00	0.00	0.00	0.00	0.00	0.00	0.00	0.00	0.00	0.00	0.00	0.00	0.00	0.00
30	f__Clostridiaceae;g__Clostridium;s__sardiniense	0.00	0.00	0.00	0.00	0.00	0.00	0.00	0.00	0.00	0.00	0.00	0.00	0.00	0.00
31	f__Clostridiaceae;g__Clostridium;s__paraputrificum	0.00	0.00	0.00	0.00	0.00	0.00	0.00	0.00	0.00	0.00	0.00	0.00	0.00	0.00
32	f__Clostridiaceae;g__Clostridium;s__perfringens	0.00	0.00	0.00	0.00	0.00	0.00	0.00	0.00	0.00	0.00	0.00	0.00	0.00	0.00
33	f__Clostridiaceae;g__Clostridium;s__neonatale	0.00	0.00	0.00	0.00	0.00	0.00	0.00	0.00	0.00	0.00	0.00	0.00	0.00	0.00
34	f__Clostridiaceae;g__Clostridium;s__saccharobutylicum	0.00	0.00	0.00	0.00	0.00	0.00	0.00	0.00	0.00	0.00	0.00	0.00	0.00	0.00
35	f__Peptostreptococcaceae;g__Terrisporobacter;s__glycolicus	0.00	0.00	0.00	0.00	0.00	0.00	0.00	0.00	0.00	0.00	0.00	0.00	0.00	0.00
36	f__Peptostreptococcaceae;g__Clostridioides;s__mangenotii	0.00	0.00	0.00	0.00	0.00	0.00	0.00	0.00	0.00	0.00	0.00	0.00	0.00	0.00
37	f__Peptostreptococcaceae;g__Paraclostridium;s__benzoelyticum	0.00	0.00	0.00	0.00	0.00	0.00	0.00	0.00	0.00	0.00	0.00	0.00	0.00	0.00
38	f__Lachnospiraceae;g__Tyzzerella;s__nexilis	0.00	0.00	0.00	0.00	0.00	0.00	0.00	0.00	0.00	0.00	0.00	0.00	0.00	0.00
39	f__Veillonellaceae;g__Veillonella;s__ratti	0.00	30.08	27.13	19.93	34.40	23.41	24.06	13.28	26.64	17.85	10.67	24.32	9.98	1.25
40	f__Veillonellaceae;g__Veillonella;s__parvula	1.49	0.00	0.00	0.00	0.00	0.00	0.00	0.00	0.00	0.00	0.00	0.14	0.00	0.00

Supplementary Table 1, continued

	Microbial species	Month 2			Month 3							Month 4			
		Day 74	Day 76	Day 82	Day 99	Day 103	Day 104	Day 108	Day 110	Day 114	Day 118	Day 122	Day 124	Day 131	Day 134
			%					%					%		
1	f__Bifidobacteriaceae;g__Bifidobacterium;s__longum subsp. Infantis	0.48	0.43	0.50	0.00	0.18	0.09	0.33	0.30	0.44	0.12	1.01	1.14	30.84	57.13
2	f__Bifidobacteriaceae;g__Bifidobacterium;s__breve	80.50	76.69	64.37	55.71	50.69	51.08	50.80	48.45	50.16	40.80	59.64	61.26	29.16	8.74
3	f__Bifidobacteriaceae;g__Bifidobacterium;s__pseudocatenulatum	0.00	0.00	0.00	0.00	0.00	0.00	0.00	0.15	0.00	0.00	0.15	0.30	0.00	0.00
4	f__Actinomycetaceae;g__Winkia;s__neuii	0.00	2.05	8.53	0.00	0.00	0.00	0.00	0.12	0.44	0.00	0.00	0.00	0.23	0.57
5	f__Enterobacteriaceae;g__Escherichia;s__coli	0.00	0.12	0.10	0.00	0.00	0.00	0.00	0.00	0.00	0.00	0.00	0.00	0.00	1.00
6	f__Enterobacteriaceae;g__Klebsiella;s__aerogenes	0.00	0.00	0.00	18.67	21.92	15.44	13.34	12.71	11.09	11.96	5.03	3.66	0.28	0.28
7	f__Enterobacteriaceae;g__Kluyvera;s__cryocrescens	0.00	0.00	0.00	9.92	12.06	8.13	7.42	7.08	6.54	6.14	2.77	2.25	0.21	0.61
8	f__Enterobacteriaceae;g__Citrobacter;s__unclassified Citrobacter	0.00	0.00	0.00	0.00	0.00	0.00	0.00	0.00	0.00	0.00	0.00	0.00	9.27	5.73
9	f__Enterobacteriaceae;g__Citrobacter;s__farmeri	0.00	0.00	0.00	0.00	0.00	0.00	0.00	0.00	0.00	0.00	0.00	0.00	1.36	0.76
10	f__Enterobacteriaceae;g__Raoultella;s__terrigena	0.00	0.00	0.00	0.00	0.00	0.00	0.00	0.00	0.00	0.00	0.62	0.94	1.47	0.00
11	f__Enterobacteriaceae;g__Citrobacter;s__freundii	0.00	0.00	0.00	0.00	0.00	0.00	0.00	0.00	0.00	0.00	0.66	1.01	1.17	0.00
12	f__Enterobacteriaceae;g__Enterobacter;s__hormaechei	0.00	0.00	0.00	0.00	0.00	0.00	0.00	0.00	0.00	0.00	0.27	0.36	0.56	0.32
13	f__Enterobacteriaceae;g__Citrobacter;s__koseri	0.00	0.00	0.00	0.00	0.47	0.85	2.67	2.11	3.29	1.85	3.23	2.22	0.13	0.00
14	f__Pasteurellaceae;g__Haemophilus;s__parainfluenzae	0.00	0.00	0.00	0.00	0.00	0.00	0.00	0.00	0.00	0.00	0.00	0.00	0.00	0.00
15	f__Streptococcaceae;g__Streptococcus;s__gallolyticus	2.08	0.70	2.38	1.59	3.35	6.85	8.26	10.42	1.63	30.07	5.05	2.24	0.73	0.97
16	f__Streptococcaceae;g__Streptococcus;s__infantis	0.43	0.19	0.07	0.50	0.13	0.32	0.14	0.68	0.33	0.19	0.27	0.64	0.52	0.40
17	f__Streptococcaceae;g__Streptococcus;s__salivarius	1.43	0.51	0.39	0.42	0.25	0.25	0.12	0.23	0.31	0.27	0.23	0.58	0.16	0.26
18	f__Streptococcaceae;g__Streptococcus;s__peroris	0.00	0.00	0.00	0.00	0.00	0.00	0.00	0.00	0.00	0.00	0.00	0.00	0.05	0.05
19	f__Enterococcaceae;g__Enterococcus;s__faecium	8.22	11.29	11.17	2.83	5.52	5.02	10.41	5.70	15.75	3.52	6.38	4.88	8.86	13.29
20	f__Enterococcaceae;g__Enterococcus;s__faecalis	2.95	4.50	5.97	2.91	3.13	2.42	4.92	3.16	5.49	1.90	7.96	5.41	5.34	3.36
21	f__Enterococcaceae;g__Enterococcus;s__casseliflavus	0.00	0.00	0.00	0.00	0.00	0.00	0.00	0.00	0.00	0.00	0.22	0.00	0.85	1.11
22	f__Streptococcaceae;g__Streptococcus;s__thermophilus	0.00	0.00	0.00	0.00	0.00	0.00	0.00	0.00	0.00	0.00	0.00	0.00	0.00	0.00
23	f__Streptococcaceae;g__Streptococcus;s__sp.	0.00	0.00	0.00	0.00	0.00	0.00	0.00	0.00	0.00	0.00	0.00	0.00	0.00	0.00
24	f__Staphylococcaceae;g__Staphylococcus;s__epidermidis	1.98	0.29	0.46	0.46	0.13	0.00	0.00	0.15	0.51	0.00	0.00	0.33	0.15	0.00
25	f__Staphylococcaceae;g__Staphylococcus;s__aureus	0.00	0.00	0.00	0.87	0.24	0.54	0.51	0.66	1.24	1.02	0.98	1.65	0.66	1.04
26	f__Staphylococcaceae;g__Staphylococcus;s__lugdunensis	0.70	0.49	0.71	0.97	0.40	1.32	0.32	0.42	0.68	0.82	0.52	0.89	0.21	0.00
27	f__Paenibacillaceae;g__Paenibacillus;s__sp.	0.00	0.00	0.00	0.00	0.00	0.00	0.00	0.00	0.00	0.00	1.17	2.01	0.00	0.00
28	f__Bacillales Family XI. Incertae Sedis;g__Gemella;s__haemolysans	0.12	0.00	0.00	0.00	0.00	0.00	0.00	0.00	0.00	0.00	0.00	0.00	0.00	0.00
29	f__Clostridiaceae;g__Hungatella;s__effluvii	0.00	0.00	0.00	0.00	0.00	0.00	0.00	0.00	0.00	0.00	0.00	0.00	0.00	0.00
30	f__Clostridiaceae;g__Clostridium;s__sardiniense	0.00	0.00	0.00	0.00	0.00	0.00	0.00	0.00	0.00	0.00	0.07	0.00	0.72	0.48
31	f__Clostridiaceae;g__Clostridium;s__paraputrificum	0.00	0.00	0.00	0.00	0.00	0.00	0.00	0.00	0.00	0.00	0.00	0.00	0.00	0.00
32	f__Clostridiaceae;g__Clostridium;s__perfringens	0.00	0.00	0.00	0.00	0.00	0.00	0.00	0.00	0.00	0.00	0.00	0.91	0.52	0.19
33	f__Clostridiaceae;g__Clostridium;s__neonatale	0.00	0.00	0.00	0.00	0.00	0.00	0.00	0.00	0.00	0.00	2.49	3.23	0.62	0.75
34	f__Clostridiaceae;g__Clostridium;s__saccharobutylicum	0.00	0.00	0.00	0.00	0.00	0.00	0.00	0.00	0.00	0.00	0.00	0.00	0.00	0.00
35	f__Peptostreptococcaceae;g__Terrisporobacter;s__glycolicus	0.00	0.00	0.00	0.00	0.00	0.00	0.00	0.00	0.00	0.00	0.00	0.00	3.08	2.42
36	f__Peptostreptococcaceae;g__Clostridioides;s__mangenotii	0.00	0.00	0.00	0.00	0.00	0.00	0.00	0.00	0.00	0.00	0.21	0.28	2.25	0.44
37	f__Peptostreptococcaceae;g__Paraclostridium;s__benzoelyticum	0.00	0.00	0.00	0.00	0.00	0.00	0.00	0.00	0.00	0.00	0.00	0.00	0.00	0.00
38	f__Lachnospiraceae;g__Tyzzerella;s__nexilis	0.00	0.00	0.00	0.00	0.00	0.00	0.00	0.00	0.00	0.00	0.00	0.00	0.00	0.00
39	f__Veillonellaceae;g__Veillonella;s__ratti	1.11	2.72	5.34	5.13	1.54	7.69	0.77	7.66	2.09	1.36	1.09	3.82	0.60	0.11
40	f__Veillonellaceae;g__Veillonella;s__parvula	0.00	0.00	0.00	0.00	0.00	0.00	0.00	0.00	0.00	0.00	0.00	0.00	0.00	0.00

Supplementary Table 1, continued

	Microbial species	Month 4				Month 5					
		Day 137	Day 142	Day 145	Day 148	Day 155	Day 157	Day 158	Day 159	Day 161	Day 163
		%				%					
1	f__Bifidobacteriaceae;g__Bifidobacterium;s__longum subsp. Infantis	60.08	60.23	60.78	52.53	49.01	64.02	59.77	55.48	65.78	53.57
2	f__Bifidobacteriaceae;g__Bifidobacterium;s__breve	3.09	2.50	5.31	2.81	4.44	3.00	2.99	6.49	8.17	3.33
3	f__Bifidobacteriaceae;g__Bifidobacterium;s__pseudocatenulatum	0.00	0.00	0.00	0.00	0.00	0.00	0.00	0.00	0.00	0.00
4	f__Actinomycetaceae;g__Winkia;s__neuii	0.23	0.35	0.24	0.00	0.21	0.29	0.42	0.00	0.00	0.06
5	f__Enterobacteriaceae;g__Escherichia;s__coli	16.15	17.81	14.74	15.14	21.02	15.28	18.79	12.11	10.24	18.20
6	f__Enterobacteriaceae;g__Klebsiella;s__aerogenes	0.00	0.00	0.00	0.00	0.00	0.00	0.00	0.00	0.00	0.00
7	f__Enterobacteriaceae;g__Kluyvera;s__cryocrescens	0.00	0.00	0.00	0.00	0.00	0.00	0.00	0.00	0.00	0.00
8	f__Enterobacteriaceae;g__Citrobacter;s__unclassified Citrobacter	0.56	0.75	0.72	0.48	1.15	0.76	0.77	0.92	0.30	0.66
9	f__Enterobacteriaceae;g__Citrobacter;s__farmeri	0.09	0.07	0.08	0.10	0.15	0.10	0.08	0.19	0.00	0.08
10	f__Enterobacteriaceae;g__Raoultella;s__terrigena	0.00	0.00	0.00	0.00	0.00	0.00	0.00	0.00	0.00	0.00
11	f__Enterobacteriaceae;g__Citrobacter;s__freundii	0.00	0.00	0.00	0.00	0.00	0.00	0.00	0.00	0.00	0.00
12	f__Enterobacteriaceae;g__Enterobacter;s__hormaechei	0.48	0.45	0.29	0.54	0.66	0.34	0.43	0.22	0.00	0.42
13	f__Enterobacteriaceae;g__Citrobacter;s__koseri	0.00	0.00	0.00	0.00	0.00	0.00	0.00	0.00	0.00	0.00
14	f__Pasteurellaceae;g__Haemophilus;s__parainfluenzae	0.00	0.00	0.00	0.00	0.00	0.00	0.00	0.00	0.00	0.00
15	f__Streptococcaceae;g__Streptococcus;s__gallolyticus	0.25	0.10	0.19	0.00	0.39	0.55	0.33	0.17	0.12	0.19
16	f__Streptococcaceae;g__Streptococcus;s__infantis	0.64	1.11	1.13	0.76	1.14	0.81	0.30	1.22	3.17	0.73
17	f__Streptococcaceae;g__Streptococcus;s__salivarius	0.10	0.00	0.21	0.13	0.28	0.12	0.10	0.11	0.51	0.11
18	f__Streptococcaceae;g__Streptococcus;s__peroris	0.07	0.15	0.11	0.08	1.21	0.96	0.26	0.56	1.90	0.54
19	f__Enterococcaceae;g__Enterococcus;s__faecium	7.15	6.44	7.06	7.53	9.26	6.39	8.24	16.79	4.20	6.21
20	f__Enterococcaceae;g__Enterococcus;s__faecalis	0.52	0.37	0.18	0.67	0.20	0.13	0.16	0.12	0.07	0.00
21	f__Enterococcaceae;g__Enterococcus;s__casseliflavus	0.42	0.89	0.00	0.91	2.61	2.05	1.46	1.76	0.00	0.51
22	f__Streptococcaceae;g__Streptococcus;s__thermophilus	0.00	0.00	0.00	0.00	0.00	0.00	0.00	0.00	0.00	0.00
23	f__Streptococcaceae;g__Streptococcus;s__sp.	0.00	0.00	0.00	0.00	0.00	0.00	0.00	0.00	0.00	0.00
24	f__Staphylococcaceae;g__Staphylococcus;s__epidermidis	0.00	0.25	0.00	0.00	0.00	0.00	0.00	0.00	0.00	0.00
25	f__Staphylococcaceae;g__Staphylococcus;s__aureus	0.90	0.73	1.48	0.47	0.50	0.23	0.21	0.30	0.69	0.36
26	f__Staphylococcaceae;g__Staphylococcus;s__lugdunensis	0.00	0.00	0.00	0.00	0.00	0.00	0.00	0.00	0.00	0.00
27	f__Paenibacillaceae;g__Paenibacillus;s__sp.	0.00	0.00	0.00	0.06	0.09	0.00	0.00	0.00	0.00	0.26
28	f__Bacillales Family XI. Incertae Sedis;g__Gemella;s__haemolysans	0.00	0.00	0.00	0.00	0.00	0.00	0.00	0.00	0.00	0.00
29	f__Clostridiaceae;g__Hungatella;s__effluvii	0.00	0.00	0.00	0.00	0.00	0.00	0.00	0.00	0.00	0.00
30	f__Clostridiaceae;g__Clostridium;s__sardiniense	0.18	0.27	0.41	0.14	0.37	0.33	0.33	0.51	0.28	3.39
31	f__Clostridiaceae;g__Clostridium;s__paraputrificum	0.00	0.00	0.00	0.00	0.00	0.00	0.00	0.00	0.00	0.00
32	f__Clostridiaceae;g__Clostridium;s__perfringens	0.35	0.18	0.18	0.18	0.17	0.35	0.20	0.12	0.32	1.95
33	f__Clostridiaceae;g__Clostridium;s__neonatale	4.17	3.00	1.58	10.36	2.07	0.60	0.66	0.19	0.48	2.20
34	f__Clostridiaceae;g__Clostridium;s__saccharobutylicum	0.00	0.00	0.00	1.07	0.00	0.00	0.00	0.00	0.00	0.00
35	f__Peptostreptococcaceae;g__Terrisporobacter;s__glycolicus	1.58	1.76	1.13	2.25	1.39	0.14	2.19	0.48	0.00	1.14
36	f__Peptostreptococcaceae;g__Clostridioides;s__mangenotii	1.94	2.09	1.86	3.55	1.14	0.31	1.71	0.10	0.15	3.43
37	f__Peptostreptococcaceae;g__Paraclostridium;s__benzoelyticum	0.00	0.00	0.00	0.00	0.00	0.00	0.00	0.00	0.00	0.00
38	f__Lachnospiraceae;g__Tyzzerella;s__nexilis	0.00	0.00	0.00	0.00	0.00	0.00	0.00	0.00	0.00	0.00
39	f__Veillonellaceae;g__Veillonella;s__ratti	1.03	0.51	2.35	0.25	2.54	3.25	0.61	2.16	3.61	2.66
40	f__Veillonellaceae;g__Veillonella;s__parvula	0.00	0.00	0.00	0.00	0.00	0.00	0.00	0.00	0.00	0.00

Supplementary Table 2. Metabolites and their chemical shifts from ^{1}H NMR data in infant feces samples*

	Metabolite	δ ^{1}H (ppm)					
1	2′-Fucosyllactose	1.23(d)	4.28(dd)	5.22(d)			
2	2-Hydroxyisovalerate	0.82(d)	0.95(d)				
3	3-Fucosyllactose	1.17(d)	5.44(d)				
4	3′-Sialyllactose	1.79(t)	3.28(t)	4.52(d)			
5	5-Aminopentanoate	1.61(m)	1.67(m)	2.22(t)	3.00(t)		
6	6′-Sialyllactose	1.74(t)	4.42(d)				
7	Acetate	1.91(s)					
8	Alanine	1.47(d)	3.78(q)				
9	Aspartate	2.66(dd)	2.80(dd)	3.89(dd)			
10	Butyrate	0.88(t)	1.54(m)	2.15(t)			
11	Cadaverine	1.46(m)	1.70(m)	3.01(t)			
12	Carnitine	3.21(s)	3.40(d)	3.43(dd)			
13	Choline	3.19(s)	3.51(m)	4.06(m)			
14	Creatine	3.02(s)	3.92(s)				
15	Formate	8.44(s)					
16	Fucose	1.19(d)	1.23(d)	3.44(dd)	4.54(d)	5.19(d)	
17	Fumarate	6.51(s)					
18	Glucose	3.23(dd)	3.39(t)	3.48(t)	4.64(d)	5.22(d)	
19	Glutamate	2.12(m)	2.33(m)	2.36(m)	3.75(dd)		
20	Glycine	3.55(s)					
21	Hypoxanthine	8.18(s)	8.20(s)				
22	Isoleucine	0.93(t)	1.00(d)	1.46(m)	3.66(d)		
23	Lactate	1.32(d)	4.11(q)				
24	Lactose	3.28(t)	3.93(d)	4.44(d)	4.45(d)	4.66(d)	5.22(d)
25	Leucine	0.94(d)	0.95(d)	1.67(m)	1.70(m)	3.73(dd)	
26	Methionine	2.11(m)	2.13(s)	2.63(t)	3.85(dd)		
27	o-Phosphocholine	3.20(s)	4.15(m)				
28	Phenylalanine	7.31(d)	7.36(t)	7.42(t)			
29	Propionate	1.04(t)	2.17(q)				
30	Pyruvate	2.36(s)					
31	Succinate	2.39(s)					
32	Taurine	3.25(t)	3.41(t)				
33	Threonine	1.31(d)	3.58(d)	4.24(m)			
34	Trimethylamine N-oxide	3.25(s)					
35	Tryptophan	7.19(m)	7.53(m)	7.74(m)			
36	Tyrosine	6.88(m)	7.18(m)				
37	Urea	5.77(s)					
38	Valine	0.98(d)	1.03(d)	2.26(m)	3.60(d)		

*Key: s, singlet; d, doublet; t, triplet; q, quartet; m, multiplet; dd, doublet of doublet.

Supplementary Table 3. Concentration of the metabolites in infant feces samples collected at months 1–5 postpartum*

	Metabolite	Month 1									Month 2								Month 3	
		Day 34	Day 36	Day 41	Day 46	Day 51	Day 53	Day 58-1	Day 58-2	Day 59	Day 61	Day 62	Day 66	Day 69	Day 72	Day 74	Day 76	Day 82	Day 99	Day 103
1	2′-Fucosyllactose (mM)	1.634	3.016	2.776	1.626	0.876	1.151	0.900	1.092	0.913	1.483	1.149	0.796	0.831	0.934	1.073	0.957	0.854	0.638	0.791
2	2-Hydroxyisovalerate (mM)	0.004	0.001	0.002	0.001	0.003	0.002	0.007	0.004	0.004	0.002	0.003	0.005	0.007	0.014	0.013	0.010	0.016	0.003	0.018
3	3-Fucosyllactose (mM)	0.718	1.039	1.354	2.456	1.539	2.022	2.562	2.797	2.323	1.891	2.157	1.593	1.834	2.031	2.471	2.416	2.352	1.573	2.109
4	3′-Sialyllactose (mM)	0.195	0.283	0.307	0.601	0.436	0.758	0.767	0.708	0.677	0.541	0.623	0.594	0.639	0.622	0.708	0.744	0.650	0.460	0.791
5	5-Aminopentanoate (mM)	0.000	0.000	0.000	0.000	0.000	0.000	0.000	0.000	0.000	0.000	0.000	0.000	0.000	0.000	0.000	0.000	0.000	0.000	0.000
6	6′-Sialyllactose (mM)	0.163	0.336	0.319	0.474	0.216	0.505	0.433	0.482	0.314	0.297	0.342	0.249	0.284	0.202	0.276	0.346	0.183	0.152	0.209
7	Acetate (mM)	0.507	1.489	3.959	6.129	1.730	5.424	4.568	2.243	4.367	3.944	6.552	8.202	4.561	2.163	1.201	1.665	1.873	3.655	1.967
8	Alanine (mM)	0.081	0.066	0.100	0.201	0.252	0.185	0.155	0.153	0.150	0.122	0.165	0.194	0.173	0.212	0.155	0.177	0.196	0.222	0.219
9	Aspartate (mM)	0.057	0.097	0.101	0.359	0.163	0.361	0.388	0.478	0.390	0.169	0.219	0.249	0.324	0.328	0.340	0.434	0.292	0.129	0.177
10	Butyrate (mM)	0.011	0.014	0.025	0.098	0.084	0.079	0.107	0.056	0.099	0.022	0.041	0.076	0.046	0.040	0.028	0.034	0.041	0.006	0.039
11	Cadaverine (mM)	0.048	0.036	0.031	0.069	0.060	0.117	0.213	0.147	0.190	0.070	0.077	0.105	0.135	0.093	0.095	0.128	0.128	0.053	0.083
12	Carnitine (mM)	0.006	0.003	0.005	0.008	0.005	0.012	0.005	0.008	0.004	0.006	0.005	0.007	0.004	0.009	0.013	0.006	0.004	0.015	0.004
13	Choline (mM)	0.015	0.009	0.004	0.009	0.037	0.009	0.009	0.007	0.008	0.018	0.013	0.027	0.014	0.018	0.008	0.007	0.010	0.042	0.009
14	Creatine (mM)	0.070	0.074	0.010	0.035	0.027	0.031	0.026	0.043	0.030	0.018	0.015	0.018	0.025	0.013	0.037	0.036	0.029	0.002	0.019
15	Formate (mM)	1.216	1.335	1.483	1.374	1.309	1.010	1.147	0.905	0.925	0.935	1.159	1.168	0.749	0.894	0.626	0.806	0.714	0.951	0.489
16	Fucose (mM)	0.414	0.103	0.460	0.358	0.280	0.351	0.207	0.388	0.272	1.229	0.979	0.209	0.355	0.381	0.380	0.444	0.474	0.102	0.320
17	Fumarate (mM)	0.001	0.000	0.002	0.002	0.000	0.005	0.002	0.002	0.002	0.002	0.003	0.003	0.004	0.003	0.003	0.002	0.004	0.001	0.002
18	Glucose (mM)	0.017	0.128	0.477	0.391	0.269	0.328	0.144	0.128	0.193	1.065	0.854	0.223	0.161	0.120	0.134	0.153	0.146	0.181	0.214
19	Glutamate (mM)	0.152	0.141	0.204	0.457	0.254	0.452	0.487	0.597	0.458	0.279	0.426	0.496	0.586	0.755	0.670	0.959	0.839	0.456	0.841
20	Glycine (mM)	0.000	0.000	0.035	0.055	0.168	0.130	0.109	0.174	0.100	0.092	0.129	0.116	0.153	0.231	0.224	0.238	0.191	0.144	0.221
21	Hypoxanthine (mM)	0.000	0.000	0.008	0.030	0.018	0.051	0.042	0.051	0.048	0.018	0.027	0.041	0.048	0.031	0.030	0.053	0.046	0.006	0.019
22	Isoleucine (mM)	0.029	0.037	0.038	0.059	0.058	0.063	0.066	0.071	0.055	0.049	0.049	0.071	0.084	0.078	0.065	0.085	0.066	0.069	0.074
23	Lactate (mM)	0.097	0.003	2.942	1.159	2.065	1.233	0.971	0.086	0.727	2.871	2.809	3.233	1.580	0.229	0.051	0.317	0.115	6.081	0.411
24	Lactose (mM)	5.275	6.211	1.000	1.115	0.707	1.038	0.948	1.039	1.006	2.025	1.541	0.730	0.839	0.706	0.911	0.796	0.867	0.334	0.408
25	Leucine (mM)	0.073	0.063	0.060	0.096	0.134	0.116	0.101	0.085	0.069	0.070	0.086	0.139	0.187	0.147	0.148	0.157	0.149	0.153	0.165
26	Methionine (mM)	0.013	0.012	0.016	0.024	0.024	0.023	0.017	0.025	0.016	0.015	0.016	0.015	0.016	0.014	0.021	0.018	0.018	0.021	0.015
27	O-Phosphocholine (mM)	0.001	0.001	0.000	0.001	0.001	0.001	0.001	0.001	0.001	0.002	0.001	0.001	0.001	0.001	0.001	0.001	0.000	0.001	0.001
28	Phenylalanine (mM)	0.033	0.025	0.022	0.033	0.042	0.041	0.035	0.051	0.039	0.023	0.032	0.047	0.067	0.044	0.050	0.059	0.063	0.043	0.048
29	Propionate (mM)	0.121	0.080	0.074	0.255	0.254	0.251	0.386	0.285	0.439	0.052	0.169	0.306	0.325	0.459	0.276	0.226	0.313	0.013	0.369
30	Pyruvate (mM)	0.157	0.022	0.081	0.247	0.088	0.139	0.065	0.046	0.085	0.154	0.169	0.231	0.096	0.068	0.032	0.069	0.074	0.078	0.082
31	Succinate (mM)	0.065	0.008	0.114	0.083	0.045	0.136	0.049	0.036	0.049	0.182	0.226	0.230	0.125	0.438	0.153	0.139	0.245	0.121	0.231
32	Taurine (mM)	0.017	0.064	0.056	0.035	0.036	0.049	0.042	0.050	0.046	0.082	0.051	0.035	0.057	0.053	0.064	0.080	0.069	0.073	0.068
33	Threonine (mM)	0.117	0.032	0.102	0.264	0.198	0.256	0.139	0.099	0.141	0.117	0.122	0.117	0.059	0.044	0.049	0.071	0.043	0.192	0.115
34	TMAO (mM)	0.008	0.015	0.016	0.022	0.015	0.020	0.028	0.033	0.026	0.019	0.013	0.022	0.009	0.028	0.028	0.021	0.005	0.009	0.002
35	Tryptophan (mM)	0.027	0.019	0.011	0.026	0.015	0.024	0.029	0.036	0.030	0.020	0.019	0.026	0.036	0.021	0.026	0.031	0.030	0.018	0.021
36	Tyrosine (mM)	0.025	0.032	0.031	0.048	0.047	0.044	0.035	0.038	0.033	0.033	0.028	0.032	0.036	0.029	0.036	0.031	0.026	0.046	0.025
37	Urea (mM)	0.182	0.172	0.191	0.234	0.216	0.211	0.212	0.244	0.188	0.199	0.152	0.218	0.198	0.217	0.150	0.163	0.187	0.155	0.155
38	Valine (mM)	0.065	0.059	0.060	0.101	0.122	0.130	0.123	0.163	0.102	0.072	0.097	0.155	0.205	0.168	0.144	0.170	0.154	0.147	0.180
39	Immunoglobulin A (g/l)	0.252	0.144	0.130	0.124	0.226	0.144	0.098	0.089	0.108	0.143	0.089	0.117	0.078	0.052	0.025	0.030	0.035	0.056	0.040
40	Lactoferrin (mg/l)	0.271	0.068	0.025	0.031	0.099	0.046	0.052	0.037	0.076	0.014	0.005	0.052	0.024	0.004	0.004	0.008	0.003	0.000	0.002
41	Lysozyme (μg/l)	0.006	0.012	0.005	0.104	0.009	0.008	0.017	0.010	0.037	0.007	0.004	0.064	0.008	0.005	0.009	0.003	0.003	0.009	0.006

*The relative concentration of each metabolite is its integral value normalized to TSP, which was added to the NMR buffer as an internal standard.

Supplementary Table 3, continued*

	Metabolite	Month 3					Month 4								Month 5					
		Day 104	Day 108	Day 110	Day 114	Day 118	Day 122	Day 124	Day 131	Day 134	Day 137	Day 142	Day 145	Day 148	Day 155	Day 157	Day 158	Day 159	Day 161	Day 163
1	2′-Fucosyllactose (mM)	0.628	0.736	0.817	0.805	0.755	0.568	0.644	0.000	0.000	0.000	0.000	0.000	0.000	0.000	0.000	0.000	0.000	0.000	0.000
2	2-Hydroxyisovalerate (mM)	0.007	0.018	0.011	0.025	0.024	0.013	0.009	0.010	0.009	0.009	0.009	0.005	0.013	0.010	0.009	0.003	0.026	0.002	0.004
3	3-Fucosyllactose (mM)	1.292	1.910	1.611	2.247	1.866	2.048	1.645	0.000	0.000	0.000	0.000	0.000	0.000	0.000	0.000	0.000	0.000	0.000	0.000
4	3′-Sialyllactose (mM)	0.425	0.687	0.568	0.683	0.770	0.042	0.000	0.000	0.000	0.000	0.000	0.000	0.000	0.000	0.000	0.000	0.000	0.000	0.000
5	5-Aminopentanoate (mM)	0.000	0.000	0.000	0.000	0.071	0.177	0.176	0.559	0.626	0.492	0.523	0.341	0.603	0.328	0.265	0.498	0.450	0.000	0.310
6	6′-Sialyllactose (mM)	0.134	0.221	0.138	0.176	0.184	0.084	0.000	0.000	0.000	0.000	0.000	0.000	0.000	0.000	0.000	0.000	0.000	0.000	0.000
7	Acetate (mM)	8.534	0.553	7.004	0.643	2.965	3.121	8.736	8.607	4.717	12.41	8.994	9.469	13.23	8.900	9.109	8.301	3.307	9.036	10.18
8	Alanine (mM)	0.248	0.174	0.233	0.231	0.253	0.195	0.260	0.291	0.341	0.405	0.229	0.339	0.432	0.473	0.419	0.268	0.648	0.239	0.353
9	Aspartate (mM)	0.107	0.113	0.098	0.082	0.130	0.218	0.128	0.450	0.750	0.501	0.646	0.471	0.670	0.294	0.170	0.583	0.591	0.124	0.349
10	Butyrate (mM)	0.012	0.020	0.012	0.025	0.047	0.069	0.064	0.363	0.184	0.242	0.356	0.201	0.443	0.112	0.025	0.325	0.123	0.013	0.112
11	Cadaverine (mM)	0.069	0.078	0.070	0.090	0.091	0.068	0.064	0.214	0.181	0.123	0.167	0.140	0.158	0.117	0.083	0.181	0.160	0.045	0.133
12	Carnitine (mM)	0.005	0.003	0.004	0.003	0.007	0.003	0.005	0.009	0.006	0.005	0.007	0.007	0.008	0.014	0.007	0.010	0.005	0.011	0.011
13	Choline (mM)	0.027	0.006	0.026	0.005	0.015	0.011	0.016	0.016	0.013	0.021	0.019	0.030	0.027	0.028	0.030	0.032	0.021	0.033	0.031
14	Creatine (mM)	0.028	0.018	0.017	0.023	0.014	0.009	0.006	0.030	0.058	0.045	0.047	0.067	0.060	0.084	0.045	0.065	0.059	0.044	0.049
15	Formate (mM)	0.708	0.457	0.732	0.438	0.655	0.650	0.949	0.876	0.712	1.351	0.746	1.108	0.880	0.793	0.635	0.446	0.938	0.747	0.837
16	Fucose (mM)	0.099	0.245	0.195	0.208	0.288	0.290	0.720	0.534	0.209	0.159	0.729	0.208	0.824	1.480	2.036	1.084	0.255	2.523	0.863
17	Fumarate (mM)	0.001	0.005	0.001	0.001	0.001	0.002	0.001	0.016	0.025	0.005	0.012	0.005	0.012	0.010	0.002	0.023	0.026	0.004	0.006
18	Glucose (mM)	0.163	0.134	0.306	0.099	0.220	0.162	0.715	0.480	0.197	0.243	0.606	0.327	0.796	0.750	0.510	0.684	0.283	0.739	0.419
19	Glutamate (mM)	0.443	0.741	0.542	0.791	0.807	0.721	0.524	0.960	0.995	0.623	0.829	0.581	1.072	0.543	0.342	1.038	0.721	0.197	0.512
20	Glycine (mM)	0.270	0.229	0.243	0.249	0.175	0.109	0.215	0.055	0.084	0.133	0.073	0.160	0.170	0.119	0.186	0.134	0.245	0.214	0.217
21	Hypoxanthine (mM)	0.005	0.032	0.009	0.055	0.017	0.022	0.011	0.016	0.007	0.011	0.005	0.008	0.006	0.005	0.008	0.007	0.011	0.007	0.009
22	Isoleucine (mM)	0.098	0.072	0.068	0.074	0.063	0.039	0.036	0.036	0.044	0.052	0.038	0.061	0.050	0.058	0.055	0.042	0.081	0.045	0.073
23	Lactate (mM)	7.617	0.039	6.313	0.035	1.555	1.940	6.159	3.226	0.591	5.560	4.339	8.311	3.837	5.942	9.531	4.307	2.380	7.981	8.756
24	Lactose (mM)	0.320	0.352	0.486	0.410	0.510	0.183	0.770	0.156	0.078	0.084	0.134	0.085	0.117	0.902	0.618	0.180	0.080	0.903	0.537
25	Leucine (mM)	0.202	0.169	0.167	0.164	0.155	0.076	0.067	0.108	0.109	0.099	0.096	0.118	0.079	0.104	0.095	0.118	0.135	0.078	0.106
26	Methionine (mM)	0.024	0.017	0.017	0.013	0.014	0.018	0.014	0.011	0.022	0.019	0.007	0.024	0.012	0.014	0.015	0.021	0.021	0.012	0.019
27	O-Phosphocholine (mM)	0.003	0.001	0.002	0.001	0.001	0.000	0.001	0.002	0.003	0.004	0.004	0.003	0.004	0.003	0.002	0.004	0.002	0.001	0.002
28	Phenylalanine (mM)	0.070	0.051	0.053	0.071	0.045	0.018	0.020	0.017	0.025	0.030	0.021	0.042	0.024	0.049	0.036	0.024	0.062	0.025	0.045
29	Propionate (mM)	0.080	0.250	0.092	0.217	0.322	0.372	0.128	0.382	0.616	0.458	0.492	0.336	0.739	0.223	0.061	0.231	0.287	0.033	0.132
30	Pyruvate (mM)	0.244	0.007	0.207	0.006	0.161	0.043	0.285	0.202	0.059	0.184	0.144	0.236	0.169	0.370	0.350	0.266	0.063	0.236	0.410
31	Succinate (mM)	0.214	0.273	0.248	0.022	0.130	0.404	0.322	0.486	0.506	0.327	0.549	0.552	0.589	0.668	0.478	0.228	1.304	0.301	0.224
32	Taurine (mM)	0.193	0.079	0.107	0.062	0.098	0.051	0.182	0.093	0.149	0.138	0.086	0.201	0.108	0.113	0.127	0.168	0.125	0.208	0.247
33	Threonine (mM)	0.120	0.040	0.081	0.016	0.071	0.088	0.081	0.044	0.120	0.152	0.112	0.155	0.155	0.134	0.173	0.160	0.191	0.105	0.125
34	TMAO (mM)	0.006	0.002	0.008	0.011	0.005	0.006	0.006	0.004	0.002	0.002	0.004	0.003	0.004	0.006	0.005	0.003	0.002	0.008	0.007
35	Tryptophan (mM)	0.024	0.014	0.011	0.031	0.009	0.014	0.013	0.022	0.018	0.019	0.014	0.017	0.020	0.010	0.014	0.015	0.017	0.009	0.017
36	Tyrosine (mM)	0.062	0.023	0.032	0.018	0.021	0.022	0.026	0.023	0.036	0.042	0.034	0.051	0.041	0.040	0.042	0.034	0.064	0.032	0.048
37	Urea (mM)	0.211	0.134	0.214	0.361	0.121	0.140	0.147	0.024	0.092	0.040	0.044	0.047	0.048	0.068	0.000	0.000	0.000	0.000	0.000
38	Valine (mM)	0.173	0.152	0.167	0.171	0.148	0.063	0.074	0.056	0.085	0.077	0.057	0.105	0.090	0.141	0.126	0.073	0.213	0.090	0.113
39	Immunoglobulin A (g/l)	0.033	0.008	0.037	0.007	0.024	0.016	0.027	0.032	0.041	0.036	0.034	0.037	0.037	0.067	0.045	0.032	0.029	0.052	0.030
40	Lactoferrin (mg/l)	0.000	0.000	0.000	0.001	0.001	0.000	0.070	0.000	0.015	0.000	0.000	0.000	0.000	0.001	0.001	0.001	0.000	0.001	0.000
41	Lysozyme (μg/l)	0.004	0.007	0.003	0.004	0.011	0.004	0.003	0.006	0.006	0.004	0.005	0.004	0.011	0.008	0.005	0.004	0.008	0.008	0.005

*The relative concentration of each metabolite is its integral value normalized to TSP, which was added to the NMR buffer as an internal standard.

Supplementary Table 4. Metabolites and their chemical shifts from ^{1}H NMR data in breast milk samples*

	Metabolite	δ ^{1}H (ppm)						
1	2′-Fucosyllactose	1.22(d)	4.24(dd)	5.22(d)	5.30(d)			
2	3-Fucosyllactose	1.17(d)	5.43(d)					
3	3′-Sialyllactose	1.79(t)	3.28(t)	4.53(d)				
4	6′-Sialyllactose	1.74(t)	4.42(d)					
5	Acetate	1.91(s)						
6	Acetone	2.22(s)						
7	Alanine	1.47(d)	3.78(q)					
8	Aspartate	2.67(dd)	2.80(dd)	3.89(dd)				
9	Betaine	3.25(s)	3.89(s)					
10	Butyrate	0.88(t)	1.54(m)	2.14(t)				
11	Caprate	0.86(t)	1.27(m)	1.53(m)	2.16(t)			
12	Caprylate	0.85(t)	1.28(m)	1.53(m)	2.16(t)			
13	Choline	3.19(s)	3.51(m)	4.06(m)				
14	Citrate	2.52(d)	2.66(d)					
15	Creatine	3.02(s)	3.92(s)					
16	Creatinine	3.03(s)	4.04(s)					
17	Formate	8.44(s)						
18	Fucose	1.21(d)	1.23(d)	4.54(d)	5.20(d)			
19	Fumarate	6.51(s)						
20	Galactose	3.48(dd)	3.98(dd)	4.57(d)	5.27(d)			
21	Glucose	3.23(dd)	3.39(t)	3.40(t)	3.48(t)	3.89(dd)	4.63(d)	5.23(d)
22	Glutamate	2.04(m)	2.12(m)	2.32(m)	2.36(m)			
23	Glutamine	2.11(m)	2.14(m)	2.42(m)	2.46(m)			
24	Hippurate	7.54(m)	7.62(m)	7.81(m)				
25	Histidine	7.06(s)	7.81(s)					
26	Hypoxanthine	8.17(s)	8.20(s)					
27	Isoleucine	0.93(t)	1.00(d)					
28	Lactate	1.32(d)	4.11(q)					
29	Lactose	3.28(t)	3.54(dd)	3.55(dd)	3.59(m)	3.95(dd)	5.22(d)	
30	Leucine	0.94(d)	0.95(d)	1.67(m)	1.70(m)			
31	Methionine	2.19(m)	2.63(t)					
32	2-Oxoglutarate	2.43(t)	3.00(t)					
33	o-Phosphocholine	3.21(s)	4.15(m)					
34	Phenylalanine	7.32(d)	7.36(t)	7.42(t)				
35	Succinate	2.39(s)						
36	Taurine	3.25(t)	3.41(t)					
37	Threonine	1.31(d)	4.25(m)					
38	Tyrosine	6.89(m)	7.18(m)					
39	Urea	5.78(s)						
40	Valine	0.97(d)	1.03(d)	2.26(m)				

*Key: s, singlet; d, doublet; t, triplet; q, quartet; m, multiplet; dd, doublet of doublet.

Supplementary Table 5. Concentration of the components in breast milk samples collected at months 1–5 postpartum*

	Component	Month 1									Month 2								Month 3	
		Day 34	Day 36	Day 41	Day 46	Day 51	Day 53	Day 58-1	Day 58-2	Day 59	Day 61	Day 62	Day 66	Day 69	Day 72	Day 74	Day 76	Day 82	Day 99	Day 103
1	2′-Fucosyllactose (mM)	1.874	1.817	1.914	1.808	1.845	1.844	1.966	1.711	1.915	1.732	1.750	1.818	1.781	1.643	1.589	1.701	1.710	1.361	1.526
2	3-Fucosyllactose (mM)	0.343	0.341	0.410	0.305	0.335	0.286	0.390	0.407	0.368	0.296	0.317	0.335	0.312	0.267	0.267	0.355	0.285	0.339	0.313
3	3′-Sialyllactose (mM)	0.142	0.149	0.145	0.144	0.139	0.138	0.131	0.151	0.150	0.131	0.139	0.139	0.138	0.121	0.114	0.149	0.132	0.126	0.141
4	6′-Sialyllactose (mM)	0.155	0.149	0.136	0.113	0.094	0.070	0.080	0.069	0.095	0.066	0.070	0.056	0.071	0.054	0.055	0.062	0.042	0.035	0.040
5	Acetate (mM)	0.010	0.016	0.008	0.013	0.007	0.019	0.019	0.006	0.017	0.009	0.014	0.009	0.008	0.007	0.010	0.016	0.007	0.009	0.012
6	Acetone (mM)	0.006	0.005	0.004	0.009	0.006	0.005	0.008	0.005	0.005	0.006	0.005	0.007	0.008	0.006	0.006	0.006	0.006	0.005	0.006
7	Alanine (mM)	0.155	0.229	0.208	0.200	0.141	0.171	0.157	0.193	0.254	0.212	0.226	0.187	0.175	0.205	0.193	0.200	0.165	0.246	0.163
8	Aspartate (mM)	0.036	0.049	0.054	0.058	0.037	0.042	0.026	0.030	0.104	0.075	0.049	0.055	0.040	0.042	0.069	0.025	0.043	0.068	0.051
9	Betaine (mM)	0.091	0.090	0.089	0.090	0.090	0.091	0.094	0.088	0.095	0.092	0.089	0.087	0.086	0.090	0.078	0.095	0.089	0.084	0.083
10	Butyrate (mM)	0.053	0.098	0.120	0.125	0.107	0.037	0.368	0.065	0.279	0.105	0.126	0.056	0.131	0.077	0.085	0.259	0.115	0.134	0.181
11	Caprate (mM)	0.087	0.202	0.287	0.260	0.186	0.073	0.527	0.123	0.568	0.195	0.212	0.101	0.160	0.112	0.142	0.410	0.204	0.221	0.308
12	Caprylate (mM)	0.158	0.329	0.353	0.374	0.212	0.100	0.475	0.144	0.439	0.249	0.241	0.087	0.161	0.121	0.157	0.375	0.236	0.182	0.280
13	Choline (mM)	0.034	0.059	0.025	0.038	0.020	0.048	0.030	0.020	0.038	0.028	0.038	0.035	0.031	0.022	0.033	0.043	0.027	0.026	0.027
14	Citrate (mM)	2.294	1.380	2.698	1.648	1.298	1.079	2.568	2.115	2.376	1.430	1.427	1.380	2.358	2.118	1.793	2.292	1.301	1.748	1.779
15	Creatine (mM)	0.035	0.050	0.033	0.034	0.037	0.041	0.035	0.032	0.041	0.041	0.039	0.040	0.039	0.033	0.042	0.042	0.033	0.029	0.044
16	Creatinine (mM)	0.064	0.043	0.059	0.066	0.056	0.058	0.053	0.047	0.065	0.069	0.062	0.058	0.056	0.056	0.055	0.062	0.047	0.042	0.055
17	Formate (mM)	0.017	0.013	0.016	0.014	0.013	0.012	0.021	0.011	0.013	0.014	0.013	0.011	0.012	0.012	0.011	0.012	0.012	0.011	0.017
18	Fucose (mM)	0.323	0.269	0.315	0.296	0.362	0.299	0.347	0.422	0.384	0.384	0.318	0.453	0.370	0.413	0.335	0.336	0.428	0.447	0.470
19	Fumarate (mM)	0.002	0.004	0.002	0.002	0.001	0.002	0.002	0.001	0.006	0.002	0.002	0.002	0.001	0.002	0.001	0.001	0.002	0.002	0.001
20	Galactose (mM)	0.761	0.721	0.737	0.735	0.752	0.770	0.797	0.684	0.817	0.797	0.743	0.753	0.774	0.762	0.642	0.809	0.787	0.700	0.675
21	Glucose (mM)	1.613	1.991	1.691	2.038	1.745	2.570	1.261	1.732	2.475	1.692	2.233	1.770	1.872	1.479	1.678	1.402	1.044	1.551	1.306
22	Glutamate (mM)	1.052	1.174	1.134	1.330	1.096	1.393	0.993	1.136	1.682	1.173	1.281	1.443	1.122	1.190	1.480	1.105	0.927	1.146	1.159
23	Glutamine (mM)	0.345	0.370	0.374	0.387	0.539	0.447	0.376	0.614	0.677	0.514	0.383	0.613	0.617	0.654	0.632	0.535	0.445	0.524	0.422
24	Hippurate (mM)	0.007	0.011	0.016	0.004	0.007	0.009	0.017	0.009	0.006	0.007	0.011	0.006	0.009	0.006	0.006	0.006	0.006	0.004	0.003
25	Histidine (mM)	0.038	0.038	0.040	0.040	0.039	0.034	0.032	0.035	0.047	0.036	0.033	0.046	0.040	0.027	0.033	0.026	0.023	0.024	0.021
26	Hypoxanthine (mM)	0.008	0.005	0.002	0.007	0.003	0.007	0.004	0.003	0.006	0.004	0.005	0.002	0.003	0.003	0.004	0.005	0.003	0.002	0.003
27	Isoleucine (mM)	0.007	0.021	0.007	0.010	0.012	0.010	0.008	0.007	0.010	0.012	0.006	0.016	0.007	0.004	0.008	0.008	0.005	0.011	0.010
28	Lactate (mM)	0.047	0.151	0.049	0.057	0.039	0.049	0.044	0.042	0.103	0.048	0.057	0.118	0.036	0.041	0.030	0.079	0.046	0.084	0.044
29	Lactose (mM)	104.2	186.7	186.6	187.8	186.2	191.0	184.0	112.2	196.1	192.8	189.2	183.3	187.0	117.7	169.7	193.2	188.3	182.3	179.5
30	Leucine (mM)	0.018	0.041	0.020	0.023	0.029	0.026	0.018	0.019	0.029	0.032	0.017	0.041	0.024	0.015	0.027	0.020	0.016	0.018	0.020
31	Methionine (mM)	0.014	0.017	0.012	0.014	0.016	0.016	0.011	0.008	0.015	0.019	0.007	0.020	0.011	0.011	0.011	0.012	0.011	0.011	0.011
32	2-Oxoglutarate (mM)	0.035	0.058	0.034	0.058	0.025	0.065	0.040	0.021	0.059	0.032	0.055	0.043	0.027	0.025	0.037	0.039	0.029	0.036	0.032
33	O-Phosphocholine (mM)	0.889	0.750	0.863	0.895	0.766	0.785	0.717	0.737	0.839	0.827	0.705	0.682	0.734	0.594	0.601	0.716	0.672	0.534	0.556
34	Phenylalanine (mM)	0.012	0.019	0.012	0.011	0.015	0.018	0.011	0.013	0.015	0.017	0.012	0.019	0.013	0.008	0.012	0.009	0.008	0.007	0.008
35	Succinate (mM)	0.009	0.009	0.007	0.009	0.006	0.010	0.007	0.006	0.009	0.010	0.009	0.009	0.007	0.007	0.008	0.007	0.007	0.006	0.006
36	Taurine (mM)	0.352	0.440	0.430	0.451	0.465	0.503	0.487	0.374	0.633	0.493	0.533	0.574	0.431	0.387	0.433	0.494	0.377	0.371	0.437
37	Threonine (mM)	0.062	0.074	0.073	0.060	0.068	0.052	0.055	0.075	0.080	0.066	0.039	0.081	0.066	0.063	0.088	0.053	0.032	0.047	0.065
38	Tyrosine (mM)	0.010	0.011	0.021	0.009	0.012	0.013	0.006	0.012	0.016	0.020	0.008	0.020	0.008	0.006	0.015	0.008	0.006	0.008	0.010
39	Urea (mM)	2.428	2.183	2.118	2.442	2.754	2.376	2.318	2.245	2.319	3.428	1.801	2.241	2.232	2.025	2.238	1.584	2.415	2.223	2.307
40	Valine (mM)	0.040	0.051	0.047	0.042	0.058	0.044	0.044	0.051	0.053	0.056	0.037	0.069	0.050	0.037	0.048	0.041	0.030	0.037	0.040
41	Fat (g/100ml)	4.1	3.9	3.4	5.2	2.3	6.7	4.2	0.9	4.1	2.4	5.3	3.2	2.5	2.9	4.4	3.6	3.0	1.7	1.9
42	Protein (g/100ml)	0.9	1.0	0.9	0.9	0.8	0.6	0.9	0.9	1.0	0.9	0.7	0.7	0.4	0.9	0.8	0.9	0.7	0.7	0.3
43	Carbohydrate (g/100ml)	8.2	8.1	8.2	8.0	8.4	8.2	8.2	8.6	8.3	8.4	8.2	8.3	7.9	8.5	8.2	8.3	8.2	8.2	8.3
44	Total Solid (g/100ml)	13.5	13.3	12.9	14.5	11.8	15.8	13.6	10.8	13.7	12.0	14.5	12.7	11.1	12.5	13.8	13.2	12.2	10.9	10.7
45	Immunoglobulin A (g/l)	0.558	0.621	0.575	0.589	0.576	0.597	0.596	0.472	0.692	0.585	0.578	0.704	0.631	0.524	0.608	0.645	0.588	0.669	0.671
46	Lactoferrin (g/l)	7.495	7.222	6.208	6.095	5.115	4.562	5.686	5.010	6.246	6.242	5.468	5.897	6.361	5.330	5.499	6.122	6.146	6.672	6.445
47	Lysozyme (g/l)	0.037	0.036	0.030	0.104	0.029	0.035	0.041	0.042	0.035	0.208	0.039	0.065	0.060	0.063	0.072	0.074	0.077	0.143	0.095

*The relative concentration of each metabolite is its integral value normalized to TSP, which was added to the NMR buffer as an internal standard.

Supplementary Table 5, continued*

	Component	Month 3					Month 4								Month 5					
		Day 104	Day 108	Day 110	Day 114	Day 118	Day 122	Day 124	Day 131	Day 134	Day 137	Day 142	Day 145	Day 148	Day 155	Day 157	Day 158	Day 159	Day 161	Day 163
1	2′-Fucosyllactose (mM)	1.777	1.854	1.899	1.958	1.499	1.591	1.344	1.801	1.648	1.544	1.622	1.761	1.527	1.696	1.802	1.487	1.534	1.576	1.744
2	3-Fucosyllactose (mM)	0.344	0.366	0.372	0.547	0.314	0.425	0.322	0.333	0.378	0.342	0.347	0.408	0.317	0.249	0.334	0.257	0.306	0.334	0.379
3	3′-Sialyllactose (mM)	0.150	0.165	0.159	0.175	0.163	0.130	0.131	0.135	0.135	0.133	0.136	0.143	0.131	0.121	0.144	0.129	0.137	0.121	0.122
4	6′-Sialyllactose (mM)	0.039	0.052	0.056	0.040	0.035	0.041	0.029	0.038	0.037	0.040	0.039	0.031	0.031	0.032	0.030	0.020	0.025	0.033	0.033
5	Acetate (mM)	0.008	0.013	0.024	0.020	0.022	0.012	0.009	0.021	0.021	0.014	0.016	0.013	0.024	0.018	0.022	0.023	0.029	0.018	0.017
6	Acetone (mM)	0.007	0.004	0.005	0.004	0.011	0.005	0.005	0.005	0.004	0.006	0.006	0.003	0.005	0.008	0.007	0.012	0.007	0.004	0.004
7	Alanine (mM)	0.136	0.199	0.242	0.221	0.156	0.218	0.216	0.266	0.166	0.244	0.183	0.220	0.278	0.231	0.159	0.105	0.181	0.407	0.196
8	Aspartate (mM)	0.032	0.069	0.064	0.060	0.027	0.097	0.049	0.153	0.025	0.055	0.048	0.060	0.045	0.075	0.022	0.021	0.074	0.091	0.047
9	Betaine (mM)	0.093	0.090	0.083	0.098	0.088	0.086	0.086	0.091	0.083	0.093	0.092	0.094	0.091	0.090	0.096	0.090	0.090	0.088	0.099
10	Butyrate (mM)	0.107	0.237	0.397	0.392	0.326	0.183	0.097	0.309	0.343	0.203	0.300	0.144	0.430	0.293	0.357	0.293	0.459	0.270	0.306
11	Caprate (mM)	0.187	0.426	0.729	0.611	0.405	0.371	0.211	0.456	0.632	0.354	0.433	0.311	0.803	0.611	0.575	0.452	0.761	0.672	0.572
12	Caprylate (mM)	0.170	0.357	0.499	0.415	0.277	0.285	0.217	0.583	0.419	0.294	0.292	0.309	0.406	0.359	0.414	0.374	0.319	0.361	0.404
13	Choline (mM)	0.027	0.045	0.059	0.042	0.039	0.033	0.027	0.053	0.052	0.040	0.071	0.047	0.070	0.037	0.067	0.057	0.067	0.055	0.056
14	Citrate (mM)	1.543	1.459	1.360	1.835	1.906	1.626	1.569	1.377	1.725	1.917	2.138	1.849	1.717	1.495	1.536	1.884	1.841	1.931	1.771
15	Creatine (mM)	0.039	0.055	0.064	0.043	0.043	0.048	0.031	0.046	0.041	0.042	0.048	0.062	0.054	0.046	0.041	0.041	0.059	0.052	0.049
16	Creatinine (mM)	0.050	0.054	0.059	0.047	0.057	0.050	0.039	0.058	0.045	0.046	0.041	0.055	0.049	0.062	0.047	0.048	0.051	0.048	0.044
17	Formate (mM)	0.016	0.016	0.018	0.015	0.017	0.019	0.018	0.017	0.020	0.014	0.016	0.015	0.014	0.016	0.011	0.017	0.014	0.012	0.013
18	Fucose (mM)	0.545	0.527	0.479	0.437	0.469	0.422	0.441	0.300	0.388	0.444	0.432	0.536	0.393	0.395	0.378	0.388	0.407	0.344	0.381
19	Fumarate (mM)	0.001	0.003	0.004	0.002	0.002	0.002	0.002	0.007	0.003	0.002	0.001	0.003	0.005	0.002	0.004	0.003	0.002	0.006	0.003
20	Galactose (mM)	0.807	0.734	0.723	0.848	0.774	0.690	0.715	0.895	0.660	0.756	0.751	0.734	0.755	0.821	0.780	0.722	0.762	0.703	0.778
21	Glucose (mM)	1.322	1.561	1.500	1.230	1.454	1.955	1.346	2.433	1.253	1.826	1.551	1.604	1.948	1.825	1.284	1.465	1.412	2.512	1.546
22	Glutamate (mM)	1.204	1.508	1.508	1.225	1.328	1.279	1.135	1.625	1.184	1.219	1.130	1.311	1.205	1.299	1.248	1.193	1.133	1.186	1.194
23	Glutamine (mM)	0.546	0.488	0.322	0.468	0.508	0.533	0.568	0.669	0.558	0.708	0.468	0.577	0.500	0.511	0.674	0.636	0.673	0.695	0.770
24	Hippurate (mM)	0.010	0.013	0.006	0.006	0.017	0.010	0.008	0.003	0.012	0.003	0.005	0.011	0.005	0.004	0.006	0.008	0.004	0.009	0.012
25	Histidine (mM)	0.024	0.032	0.032	0.026	0.027	0.021	0.021	0.029	0.030	0.028	0.021	0.028	0.025	0.036	0.034	0.030	0.031	0.021	0.029
26	Hypoxanthine (mM)	0.003	0.004	0.002	0.003	0.004	0.003	0.002	0.004	0.004	0.003	0.003	0.004	0.005	0.006	0.006	0.006	0.004	0.005	0.006
27	Isoleucine (mM)	0.013	0.010	0.032	0.009	0.010	0.008	0.006	0.012	0.010	0.007	0.012	0.015	0.009	0.007	0.011	0.009	0.014	0.006	0.006
28	Lactate (mM)	0.025	0.048	0.157	0.049	0.066	0.046	0.054	0.068	0.072	0.052	0.055	0.072	0.171	0.066	0.080	0.062	0.074	0.130	0.048
29	Lactose (mM)	190.2	187.9	172.5	203.9	188.3	185.3	179.7	171.1	184.4	194.5	195.1	89.0	198.6	189.0	197.4	186.5	198.1	205.0	122.1
30	Leucine (mM)	0.030	0.033	0.059	0.023	0.027	0.020	0.016	0.028	0.029	0.024	0.024	0.031	0.020	0.020	0.030	0.027	0.032	0.015	0.024
31	Methionine (mM)	0.013	0.016	0.015	0.012	0.013	0.012	0.010	0.012	0.010	0.012	0.013	0.014	0.009	0.011	0.015	0.013	0.014	0.009	0.014
32	2-Oxoglutarate (mM)	0.026	0.039	0.074	0.040	0.052	0.031	0.036	0.022	0.061	0.041	0.038	0.046	0.075	0.045	0.062	0.081	0.056	0.079	0.049
33	O-Phosphocholine (mM)	0.668	0.629	0.615	0.614	0.623	0.609	0.517	0.530	0.538	0.614	0.572	0.523	0.615	0.756	0.645	0.569	0.606	0.561	0.614
34	Phenylalanine (mM)	0.011	0.015	0.023	0.014	0.011	0.011	0.008	0.015	0.015	0.012	0.012	0.014	0.009	0.012	0.015	0.011	0.020	0.010	0.014
35	Succinate (mM)	0.006	0.007	0.008	0.006	0.009	0.005	0.006	0.004	0.006	0.005	0.005	0.005	0.006	0.007	0.009	0.010	0.008	0.009	0.009
36	Taurine (mM)	0.468	0.685	0.676	0.479	0.430	0.497	0.379	0.648	0.469	0.585	0.533	0.618	0.575	0.509	0.564	0.620	0.637	0.613	0.589
37	Threonine (mM)	0.076	0.084	0.091	0.049	0.069	0.076	0.042	0.074	0.072	0.073	0.064	0.072	0.045	0.069	0.090	0.064	0.080	0.066	0.082
38	Tyrosine (mM)	0.008	0.015	0.024	0.012	0.008	0.005	0.005	0.010	0.011	0.009	0.009	0.013	0.006	0.010	0.011	0.006	0.015	0.005	0.011
39	Urea (mM)	2.753	2.439	2.677	2.498	1.637	2.163	1.936	1.514	1.922	1.921	1.900	2.259	2.405	2.387	2.094	2.498	2.000	1.737	1.947
40	Valine (mM)	0.055	0.051	0.074	0.034	0.047	0.042	0.028	0.042	0.045	0.040	0.041	0.051	0.034	0.035	0.043	0.039	0.044	0.025	0.045
41	Fat (g/100ml)	1.2	3.6	6.7	4.0	3.2	2.0	1.0	3.4	4.1	1.8	3.3	1.9	3.7	2.9	3.9	3.0	4.1	2.9	3.4
42	Protein (g/100ml)	0.7	0.7	0.6	0.6	0.7	0.7	0.7	0.5	0.7	0.7	0.6	0.6	0.7	1.0	0.8	0.9	0.7	1.0	0.9
43	Carbohydrate (g/100ml)	8.4	8.2	7.7	8.3	8.2	8.4	8.5	6.9	8.0	8.1	8.0	8.3	7.9	8.1	8.0	8.2	8.0	8.3	8.3
44	Total Solid (g/100ml)	10.6	12.9	15.3	13.2	12.4	11.4	10.5	11.1	13.2	11.0	12.2	11.1	12.7	12.2	13.0	12.2	13.0	12.3	12.9
45	Immunoglobulin A (g/l)	0.553	1.057	1.181	0.719	0.759	0.585	0.484	0.669	1.210	0.703	0.656	0.733	0.719	0.684	0.640	0.596	0.636	0.691	0.683
46	Lactoferrin (g/l)	5.123	6.829	9.000	5.042	7.545	5.165	4.523	5.328	10.38	4.950	5.196	6.685	6.253	7.122	5.862	14.81	5.332	5.344	5.247
47	Lysozyme (g/l)	0.090	0.104	0.105	0.102	0.169	0.094	0.072	0.060	0.218	0.108	0.136	0.125	0.094	0.106	0.141	0.295	0.157	0.082	0.081

*The relative concentration of each metabolite is its integral value normalized to TSP, which was added to the NMR buffer as an internal standard.
